# Budget impact of upadacitinib in patients with moderate to severe rheumatoid arthritis in Argentina

**DOI:** 10.17843/rpmesp.2024.412.12934

**Published:** 2024-06-17

**Authors:** Natalia Espinola, Anastasia Secco, Dario Balan, Diego Kanevsky, Guido Calvi, Pierre Morisset, Ariel Bardach, Federico Augustovski

**Affiliations:** 1 Instituto de Efectividad Clínica y Sanitaria, Ciudad de Buenos Aires, Argentina. Instituto de Efectividad Clínica y Sanitaria Ciudad de Buenos Aires Argentina; 2 AbbVie Argentina, Buenos Aires, Argentina. AbbVie Argentina Buenos Aires Argentina

**Keywords:** Budget impact, Rheumatoid arthritis, Argentina, biological drugs, upadacitinib

## Abstract

**Objectives.:**

To analyze the budget impact of upadacitinib (UPA) 15 mg + methotrexate (MTX) for the treatment of moderate-to-severe rheumatoid arthritis (RA) in patients with an inadequate response to conventional disease-modifying antirheumatic drugs (cDMARD-IR) from the perspective of social security and the private health sector in Argentina.

**Materials and methods.:**

A budget impact analysis model was developed for a hypothetical cohort of 100,000 adults with health insurance coverage who were diagnosed with RA over a 5-year time horizon. The model parameters were obtained through literature review and validated by local experts. The costs are expressed in 2024 US dollars (USD).

**Results.:**

The introduction of UPA 15 mg + MTX for the treatment of moderate-to-severe RA and cDMARD-IR resulted in minimal increase, with a five-year total cumulative incremental cost of USD 1,855 for social security and USD 1,812 for the private health sector, representing 2% of the total budget. The acquisition cost of UPA was the most influential variable in the sensitivity analysis.

**Conclusions.:**

The introduction of UPA 15 mg + MTX for the treatment of moderate-to-severe RA and cDMARD-IR can provide an effective treatment option with a minimal increase in costs for the healthcare system in Argentina, which is especially important in developing countries where health system budgets are more limited. Providing evidence-based estimates is a valuable tool for informing healthcare policies and can help policymakers make informed decisions about the allocation of healthcare resources to improve patient outcomes while also managing costs.

## INTRODUCTION

Rheumatoid arthritis (RA) is a chronic autoimmune rheumatic disease of unknown etiology characterized by polyarticular and symmetrical inflammation of the small and large joints with potential systemic involvement. RA progresses and may lead to joint destruction, disability, and an increased risk of mortality ^1^. In Argentina, its estimated prevalence is 0.94% (95% CI 0.86%-1.02%), and the annual incidence rate is 18.50 (95% CI 16.70-20.40) per 100,000 people; 25.20 (22.40-28.00) in women and 8.80 (6.80-10.80) in men ^2^^-^^4^. There is no cure for RA; treatment is aimed at controlling synovitis, preventing joint damage, reducing comorbidities and maintaining function and quality of life ^5^^-^^7^. The treatment strategy depends on disease activity, response to previous therapies, comorbidities, patient preferences, and concerns about access and cost and is based on the use of disease-modifying antirheumatic drugs (DMARDs), primarily conventional DMARDs (cDMARDs). Methotrexate (MTX) is the most frequently used cDMARD. In patients with an inadequate response to initial treatment, biologic DMARDs (bDMARDs) are administered, including anti-tumor necrosis factor agents (anti-TNFs), abatacept, tocilizumab, sarilumab, and rituximab. bDMARDs are intended for parenteral administration, either subcutaneously or intravenously. Janus kinase inhibitors or anti-JAKs are a new class of recently introduced drugs that include baricitinib, tofacitinib, and upadacitinib; these can be administered orally and have a shorter half-life than bDMARDs ^6^^-^^8^.

In Argentina, the use of cDMARDs, most commonly MTX, as first-line therapy is standard practice ^5^^-^^9^. The anti-JAK upadacitinib has shown efficacy and safety in patients with cDMARD-IR and bDMARD-IR; in Argentina, it was approved in March 2020 ^10^^-^^14^. As a single-agent therapy, upadacitinib also showed its superiority compared with MTX in MTX-naive patients with early RA, and inadequate response to MTX patients, and when compared with adalimumab and abatacept ^14^^-^^17^. Treatment strategies for patients with moderate-to-severe RA and cDMARD-IR are associated with high costs. In Argentina, the average annual total cost of AR without biologics was USD 3,093 in 2002. Hospitalizations represented 73% of the total direct medical costs, while drugs and outpatient procedures accounted for 16% and 8% of total direct medical costs, respectively ^18^.

In recent decades, healthcare systems have experienced a growing increase in spending, which has led to resource-constrained settings such as Argentina requiring tools for the efficient allocation and prioritization of healthcare resources, while improving patient outcomes. The Latin America Policy Forum 2018 concludes that one of the essential dimensions for decision-making in budget impact analysis, i.e. it is essential to analyze the affordability of incorporating new technologies ^19^.

In Argentina, the expanded use of biologics for patients with RA from 2012 to 2022 could result in cumulative net cost savings of USD 495 billion in 10 years (exchange rate: 4.75 Argentine pesos (ARS)/United States Dollars (USD)) ^20^. However, no economic evaluation studies of upadacitinib have been conducted in Argentina. Our aim was to assess the budget impact of introducing upadacitinib for the treatment of RA over a 5-year time horizon from the perspective of social security and the private health sector in Argentina.

**KEY MESSAGES**

Motivation for conducting the study. Rheumatoid arthritis (RA) is a disease that hasn’t cure, so it’s important to know the budget impact of treatment with upadacitinib (UPA) 15 mg + methotrexate (MTX) in patients with moderate to severe RA who didn’t respond well to conventional antirheumatic drugs.

Main findings. UPA + MTX would entail a minimal increase in costs for the healthcare system in Argentina, potentially making this effective treatment option more accessible to patients with RA. Access to this treatment can improve the outcome of patients with RA.

Public health implications. In resource-constrained settings such as Argentina, providing evidence-based cost estimates can help healthcare managers allocate resources efficiently while improving patient outcomes. This study provides evidence to inform healthcare policies and decisions regarding the inclusion of UPA + MTX in treatment guidelines or formularies for RA management.

## MATERIALS AND METHODS

### Analytical Framework

**Figure 1:**
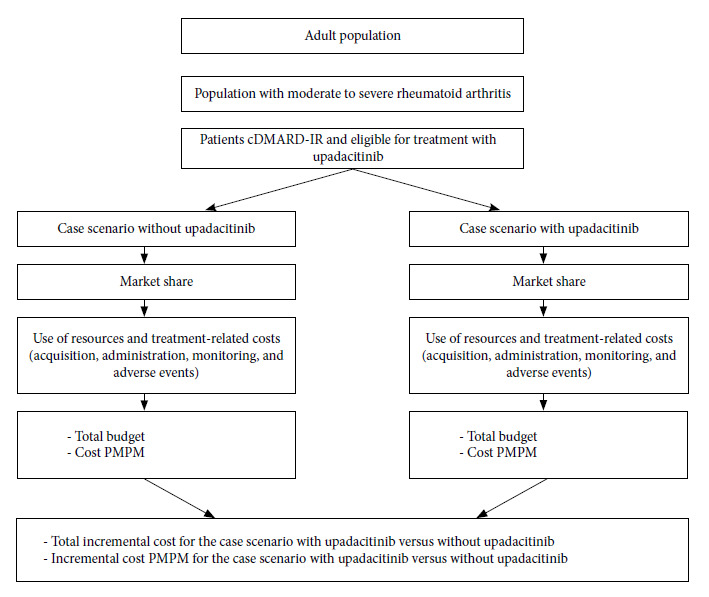
Analytical model

We developed a prevalence-based model for the budget impact analysis using a static approach with open cohort at steady state, in Excel (Figure 1), following the guidelines in models by Mauskopf et al (2017) ^21^. The analytical structure of the model consisted of four main components that, combined, resulted in the estimation of the budget impact for the health funder in the current state (without upadacitinib) and in the upadacitinib incorporation strategy for five budget years. The components were 1) the estimation of the population that would receive the technology; 2) the drug market share (current state and upadacitinib incorporation strategy); 3) estimation of the drug acquisition, administration, and routine care costs; and 4) the costs associated with the management of adverse events. The model was developed following the recommendations of the International Society for Pharmacoeconomics and Outcomes Research principles of good practices ^22^.

We considered the current market without upadacitinib and a hypothetical market with the drug being introduced over a 5-year time horizon and calculated the budgetary effects resulting from its incorporation. Upadacitinib 15 mg + methotrexate was used as the intervention drug. The drugs considered in the scenario without upadacitinib (comparators) were abatacept (subcutaneous and intravenous), adalimumab, baricitinib, certolizumab pegol, etanercept, golimumab, infliximab, rituximab, sarilumab, tocilizumab, tofacitinib (all in combination with methotrexate), and intensive cDMARDs (including hydroxychloroquine 6.5 mg/kg/d, prednisone 7.5 mg/d, sulfasalazine 2 g/d, and methotrexate 20 mg/wk). The comparators were selected by the work team based on the therapeutic options available in the Argentine context and approved by the National Administration of Medicines, Food and Medical Technology (ANMAT) for their commercialization.

The eligible population was estimated as the proportion of a theoretical cohort of 100,000 adults with moderate to severe RA and cDMARD-IR, based on potential incidence and prevalence rates across the country, as described in the literature. The analysis was performed from the perspectives of the social security sector and private health sector over a 5-year time horizon based on the recommendations of the economic evaluation guides ^23^. The results were not adjusted for discount rates or inflation ^23^.

The key model assumptions were as follows: a) the incidence and prevalence rates were constant over the analysis period; b) cases lost to death, treatment discontinuation, or disease progression were assumed to be captured in prevalence rates; c) the percentage of patients with moderate to severe RA eligible for biologic therapy remained constant in each time period; and d) the drug acquisition costs assumed that the cohorts of incidence and prevalence patients were treated for a full year, as the time when both cohorts met and began treatment was unknown.

After calculating the population that could be treated in the scenario without upadacitinib and in the scenario with upadacitinib (by multiplying the target population by the market share of each drug), the total cost of treatment during the 5-year projection was estimated to receive the total budget impact of each scenario. To calculate the net budget impact of upadacitinib, the budget that did not include upadacitinib was subtracted from the budget with upadacitinib (Figure 1). This study presented the absolute differential costs, participation in annual costs, and differential costs per member per month by the health sector. To assess the robustness of the results, a one-way sensitivity analysis was performed by changing the following parameters by ±20%: upadacitinib price, tofacitinib price, abatacept IV price, percentage of patients who did not respond to conventional drugs, percentage of moderate-to-severe RA, market share of upadacitinib, and incidence rate using the 95% CI values (16.7% - 20.4%).

### Target Population

Epidemiological data to estimate the patient population eligible for treatment were obtained from a literature review and validated through a Delphi panel of local experts (Supplementary Material shows questionnaire responded by experts). As a result of the consensus process, it was estimated that the RA prevalence in adults in Argentina was 0.80%, closely matching the prevalence in a well-designed study conducted in Luján, Argentina (0.94% [95% CI: 0.86-1.02]) ^2^. Although our results did not provide a representative epidemiological picture, they were a reasonable approximation of the disease prevalence in Argentina ^2^. We considered an annual incidence rate of 18.50 per 100,000 adult people (95% CI 16.70-20.40), as reported in a study performed by a leading local healthcare organization ^3^. Experts estimated that percentage of adult patients with moderate to severe RA was 64%, whereas 45% of these patients had cDMARD-IR and were eligible for treatment with upadacitinib ^24^. The main epidemiological parameters of the model are summarized in Table 1.

**Table 1:** Main epidemiologic parameters

Parameters		Mid Value	Lower Value	Higher Value	Source
Prevalence of RA in Argentina; cases per 1,000 people		0.8	0.3	1.2	Scublinsky et al^2^ + experts
Incidence of RA in Argentina, cases per 100,000 people		18.5	16.7	20.4	Di et al^3^
Distribution of RA cases by severity, %					
	Mild	36	-	-	Experts
	Moderate	33	-	-	Experts
	Severe	31	-	-	Experts
Patients with moderate to severe RA cDMARD-IR and eligible to receive upadacitinib, %		45	36	54	Arturi et al^22^ + experts

cDMARD-IR, inadequate response to conventional disease-modifying antirheumatic drugs; RA, rheumatoid arthritis.

### Costs

The analysis was performed from the perspectives of social security and the private health sector. In Argentina, the healthcare system comprises three subsystems: social security, private health sector, and public health sector. Social security and the private health sector together account for 65% of the Argentine population and nearly 54% of the total health expenditure (45% for social security and 9% for private health sector) ^25^. Only direct medical costs, including those of drug acquisition, drug administration, disease monitoring, and adverse events, were considered in this analysis.

The micro-costing method was used to estimate the costs of health events. This method involves identifying health resources, rates of use, and quantity. The unit costs of health resources were identified for each subsector (social security and private sector). The expected total cost for each event resulted from the sum of the products of these three components (rates of use, quantities, and unit costs). The identification and measurement of health resources (amounts and rates of use) resulted from the literature review and the Delphi process. The value of the unit costs was obtained from the Unit Cost Basis of the Institute for Clinical Effectiveness and Health Policy ^26^. The costs were expressed in 2024 USD. The mean nominal exchange rate in January 2024 was ARS 831.35 to USD 1 ^27^.

Drug acquisition costs were derived from official national price lists ^28^^,^^29^. We used ex-factory prices for the analysis, considering a 57% discount on the retail price approximately ^30^. The doses and administration schedules for each biological agent were provided by drug inserts, local guides and a rheumatology expert ^5^^-^^9^. In Argentina, upadacitinib was approved by the National Administration of Drugs, Food, and Medical Technology (ANMAT; Spanish acronym) in December 2019.

Table 2 summarizes the annual total cost of drugs included in treatments, estimated using the ex-factory price, and taking units received during one year by a patient, with the ex-factory price per unit. In the base-case scenario, the annual acquisition cost of upadacitinib was USD 47 per unit of 15 mg (USD 3.15/mg).

**Table 2:** Dosing, ex-factory price and annual drug acquisition cost per patient a

Treatment	Vial Size, mg	Route of Administration	Dose	Units/Year	Ex-Factory Price/mg, USD	Annual Cost per patient, USD
UPA	15	Oral	15 mg/day	365	3.15	17,251
ABT	250	IV	750 mg at day 0, 15, 30, and monthly thereafter	42	2.72	28,588
ABT	125	SC	125 mg/week	52	3	19,512
ADA	40	SC	40 mg/2 weeks	26	13,36	13,898
BRC	4	Oral	4 mg/day	365	6.91	10,083
CTZ	200	SC	400 mg at day 0, 15, 30, and monthly thereafter	28	3.17	17,742
ETN	50	SC	50 mg/week	52	6.4	16,633
GOL	50	SC	50 mg/month	12	30.76	18,455
IFX	100	IV	3 mg/kg at day 1, week 2, week 6 and each 8 weeks	16	3.92	6,275
MTX	10	Oral	20 mg/week	104	0.06	58
Intensive cDMARD	-	-	^b^	-	-	630
RTX	500	SC	2000 mg each 9 months	8	4.88	19,513
SRL	200	SC	200 mg each 2 weeks	26	2.88	14,969
TCZ	200	IV	8 mg/kg/month	36	3.28	23,581
TCZ	162	SC	162 mg/week	52	2.75	23,162
TFC	5	Oral	10 mg/day	730	3.35	12,220

ABT=abatacept; ADA=adalimumab; USD=United States dollars; BRC=baricitinib; cDMARD=conventional disease-modifying antirheumatic drug; CTZ=certolizumab pegol; ETN=etanercept; GOL=golimumab; IFX=infliximab; IV=intravenous; MTX=methotrexate; RTX=rituximab; SC=subcutaneous; SRL=sarilumab; TCZ=tocilizu-mab; TFC=tofacitinib; UPA= upadacitinib.

a Costs are shown in 2024 US dollars.

b Intensive cDMARD considers hydroxychloroquine 6.5 mg/kg/d, prednisone 7.5 mg/d, sulfasalazine 2 g/d and methotrexate 20 mg/wk.

Source: Author’s elaboration. Drug price information was obtained from. ^28^^,^^29^

On the other hand, the estimated cost of intravenous administration was USD 113 for the social security sector and USD 190 for the private health sector; the cost of subcutaneous administration was USD 1.87 and USD 2.61 in the social security and private sectors, respectively; oral administration was associated with no costs. In turn, the annual monitoring cost for patients before treatment was estimated at USD 160.28 for social security and USD 198.05 for the private sector; the cost for the first 6-month treatment period was USD 58.25 and USD 71.21, for social security and the private sector, respectively; and the monthly cost was USD 8.46 and USD 10.27 for the social security and private sector, respectively. Table S1 shows the resources identified in this category, their frequency, and the unit cost for both sectors. Severe infections were considered the most prevalent treatment-associated adverse event (AE). AE rates were derived from the NICE submission of baricitinib using data from the RA-BEAM study. Adverse event rates were extrapolated to the class of drugs. The rates used in the model are presented in Table S2 in the Appendix. For costs related to AE, the cost of pneumonia was considered representative of all costs associated with serious infections and was estimated at USD 1,605 for social security and USD 2,267 for the private sector.

### Distribution of treatment schedules

Parameters related to the distribution of the rate of utilization of biologics and anti-JAKs were determined by an expert panel, based on the treatment patterns for biologic therapy in patients with RA in a 2018 study conducted in Argentina ^31^, and a literature review ^4^. Based on international guidelines, treatment with bDMARDs or anti-JAKs was preferred over combination therapy with a Cdmard ^6^. However, combination therapy with cDMARDs is used in everyday practice in Argentina because of patients’ good response to treatment and limited access to some of the drugs. As a result, these agents were accepted as treatment options and presented to panelists who confirmed this practice and agreed on a market share. The same was applied for the scenario with upadacitinib. Table 3 shows the percentage distribution of the treatment schedules. We assumed that, in the current scenario without upadacitinib, market shares were constant over five years (Table 3).

**Table 3:** Distribution of treatment schedules for patients with inadequate response to cDMARDs: Case scenarios with and without upadacitinib.

Treatment Schedule	Without Upadacitinib, %	With Upadacitinib, %				
	Years 1-5	Year 1	Year 2	Year 3	Year 4	Year 5
UPA 15 mg + MTX	0	3,25	6,75	10,74	14.85	18,13
ABT IV + MTX	1,27	1,08	0,87	0,64	0.38	0,18
ABT SC + MTX	3,81	3,62	3,41	3,17	2,9	2,66
ADA + MTX	13,71	13,51	13,31	13,06	12,7	12,37
BRC + MTX	1,02	0,82	0,62	0,38	0,13	0,00
Intensive cDMARD^a^	28,68	28,49	28,28	28,01	27,52	27,04
CTZ + MTX	8,12	7,93	7,72	7,48	7,17	6,89
ETN + MTX	22,72	22,33	21,92	21,42	20,99	20,57
GOL + MTX	2,54	2,35	2,14	1,9	1,64	1,42
IFX + MTX	1,27	0,89	0,47	0,13	0,00	0,00
RTX + MTX	3,43	3,04	2,63	2,16	1,89	1,67
SRL + MTX	1,02	0,82	0,62	0,38	0,13	0,00
TCZ IV + MTX	1,02	0,82	0,62	0,38	0,13	0,00
TCZ SC + MTX	3,81	3,62	3,41	3,17	2,9	2,66
TFC + MTX	7,61	7,42	7,22	6,97	6,67	6,4
Total	100,00	100,00	100,00	100,00	100,00	100,00

ABT=abatacept; ADA=adalimumab; BRC=baricitinib; cDMARD=conventional disease-modifying antirheumatic drug; CTZ=certolizumab pegol; ETN=etanercept; GOL=go-limumab; IFX=infliximab; IV=intravenous; MTX=methotrexate; RTX=rituximab; SC=subcutaneous; SRL=sarilumab; TCZ=tocilizumab; TFC=tofacitinib; UPA=upadacitinib.aIntensive cDMARDs included hydroxychloroquine 6.5 mg/kg/d, prednisone 7.5 mg/d, sulfasalazine 2 g/d, and methotrexate 20 mg/wk.Source: Author’s elaboration-based data extracted from the expert panel.

### Decision Rule -budgetary impact threshold

Our study employed the methodology utilized by the National Commission for Health Technology Assessment and Clinical Excellence of the Ministry of Health (CONETEC, acronym in Spanish) in the country to estimate a threshold of high budgetary impact. This approach is grounded in the study of Pichón-Riviere and colleagues, which is particularly relevant for countries lacking their own estimates ^32^. The reference value of the high budgetary impact threshold is estimated at 0.00016 health spending units (0.00008-0.00024). The estimation of the threshold of high budget impact in Argentina for 2023 was made using the reference value and estimation of total health expenditure. This latter is estimated using data from Gross Domestic Product (GDP) and total population of Argentina, and the average of the last ten available years of healthcare expenditure as a percentage of GDP ^33^^,^^34^. Accordingly, it was estimated that the threshold of high budget impact is USD 0.010 PMPM (USD 0,005 -0.015) for the health system.

## RESULTS

The budget impact of introducing upadacitinib 15 mg + MTX over a 5-year period for patients with moderate-to-severe RA and cDMARD-IR from the perspective of social security and the private health sector is shown in Table 4. For a hypothetical cohort of 100,000 adults with health insurance coverage, 23 patients with moderate to severe RA per year would be eligible for biologic therapy. Introducing upadacitinib, at USD 3.15 per mg, the cost of the treatment of moderate to severe RA increased slightly. In the case of the social security sector, the total annual cost of treatment without upadacitinib was USD 17,460 versus USD 17,568 for year 1 and USD 18,121 for year 5 with the introduction of upadacitinib. For the private health sector, the total annual cost of treatment without upadacitinib was USD 18,223 versus USD 18,328 for year 1 and USD 18,870 for year 5 with the introduction of upadacitinib.

**Table 4:** Base case results: annual costs with and without upadacitinib, absolute differential costs, participation in annual cost and differential costs per member per month from social security and the private health sector ^a^

	Total Annual Cost without Upadacitinib, USD	Total Annual Cost with Upadacitinib, USD	Absolute Difference, USD	Participation on annual cost, %	Differential costs PMPM, USD
	A	B	C=(B-A)	C/A	D*
Social security					
Year 1	17,460	17,568	108	0,62	0,00009
Year 2	17,460	17,648	223	1,28	0,000186
Year 3	17,460	17,812	352	2,02	0,000293
Year 4	17,460	17,972	512	2,93	0,000427
Year 5	17,460	18,121	660	3,78	0,00055
Total/Mean	87,302	89,157	371	2,13	0,000309
Private health sector					
Year 1	18,223	18,328	105	0,58	0,000087
Year 2	18,223	18,441	218	1,19	0,000181
Year 3	18,223	18,566	343	1,88	0,000286
Year 4	18,223	18,723	500	2,74	0,000417
Year 5	18,223	19,869	646	3,55	0,000539
Total/Mean	91,114	92,926	362	1,99	0,000302

USD=United States dollars; PMPM=per member per month.

a Costs are shown in 2024 US dollars.

* The annual cost differential per member per month was obtained by dividing the annual cost differential (C) by 100,000, and the result was divided by 12 months.

The introduction of upadacitinib was associated with a marginal annual net budget impact of an average 2% increase over a 5-year period. The net budget effect per member per month (PMPM) was USD 0.0005 at year 5. If a social security payer reimbursed treatment with upadacitinib, the average budget increase would be USD 0.0003 PMPM. The results for the private health sector are comparable, with a marginal cost increase. The analysis of the budget impact per cost category and treatment is presented in the Supplementary Material Tables S2 and S3.

**Figure 2:**
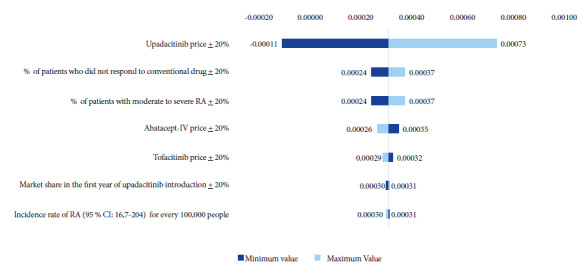
Deterministic sensitivity analysis: Average budget impact PMPM from the social security perspective, USD

The results of the deterministic sensitivity analysis for upadacitinib 15 mg + MTX in patients with moderate-to-severe RA and cDMARD-IR are shown in Figure 2. Several parameters associated with increased uncertainty were selected, and the unit price of upadacitinib showed the highest variability. In the social security sector, a 20% increase in the upadacitinib price would result in an incremental annual net budget of USD 0.0007 PMPM, accounting for an average of 5% of the total budget for the 5-year period, whereas a 20% decrease in the drug price would result in annual net budget cost savings of USD -0.0001 PMPM, accounting for an average savings of 0.75% of the annual budget. Minor variations were observed for the other parameters. For the private sector, the outcomes of the deterministic sensitivity analysis were comparable.

## DISCUSSION

With an estimated cost of USD 47 per unit of 15 mg, the introduction of UPA + MTX would entail a minimal increase in costs for the healthcare system in Argentina, potentially making this effective treatment option more accessible to patients with RA. Complementary, the budget impact results did not exceed the estimated high threshold for budgetary impact in Argentina. Thus, our findings can provide budget impact evidence to payers who are considering incorporating upadacitinib onto their formulary to treat RA, as well as a tool to inform value-based price negotiations or risk-sharing agreements. The acquisition cost of RA drugs accounted for the main direct medical cost, which is consistent with other research conducted in the local setting ^4^.

We calculated the net budget effect per member per month (PMPM), which represents the additional cost or savings for each member covered by a health plan. For example, if an institution with 2 million members was part of the social security system reimbursing for upadacitinib treatment, the total average increase across all 2 million members would be USD 0.0003 PMPM multiplied by 2 million members, which equals USD 600 in monthly increase. Introducing this new treatment option represents a minimal increase in costs for a large health plan while providing an additional effective therapy for rheumatoid arthritis patients.

According to clinical guidelines, patients with cDMARD-IR and poor prognostic factors may receive several biologics and other molecules (anti-JAKs) ^5^^-^^7^. For patients with no poor prognostic factors but an inadequate response, guidelines recommend switching to or adding another cDMARD (combination cDMARD, prescribing a bDMARD or an anti-JAK) ^5^^-^^7^. We found no economic evaluations of other anti-JAK molecules for the treatment of RA in Argentina. A 2021 evaluation of the economic effects of upadacitinib in patients with RA performed in the US showed that upadacitinib combination therapy versus tofacitinib combination therapy and upadacitinib monotherapy versus methotrexate monotherapy were associated with significantly lower direct medical costs ^35^. An analysis of the cost minimization and budget impact of upadacitinib in the treatment of RA in Brazil’s Unified Health System (SUS) over a 5-year time horizon showed that upadacitinib had lower costs than baricitinib and that it had the lowest cost among biologic DMARDs available in SUS; in comparison to our analysis, the introduction of upadacitinib resulted in net savings in Brazil ^36^.

A cost-effectiveness study of the treatment for patients with RA and cDMARD-IR and patients with inadequate response to anti-TNF bDMARDs demonstrated that tofacitinib was frequently used for patients with cDMARD-IR and that it was associated with cost-savings (€ -337,489/quality-adjusted life year gained) in patients with an inadequate response to anti-TNF ^37^. In the US, another study showed that a treatment strategy using tofacitinib resulted in reduced costs and an improved quality of life when compared with other treatment strategies ^38^. An evaluation of the economic effects of the anti-Jak molecule tofacitinib, introduced after an inadequate response to MTX or one or two anti-TNFs, showed that tofacitinib, as a second- or a third-line therapy, was associated with lower costs than tofacitinib in the fourth-line setting following MTX and two anti-TNFs sequentially ^39^. In a 2020 analysis of the budget impact and the cost per additional responder for baricitinib, another anti-JAK molecule, for the treatment of moderate to severe RA in patients with an inadequate response to anti-TNFs, baricitinib was a more cost-effective option than other DMARDs with comparable efficacy in patients with an inadequate response to anti-TNFs ^40^.

Our study has several limitations that should be acknowledged. Our results are based on projections of the market share. Additionally, the use of a static model may have oversimplified the natural history of the disease; however, our evaluations were structured based on the recommendations of Mauskopf *et al*^21^. The lack of local epidemiologic data was another limitation; therefore, local experts were asked to validate the selected parameters. In addition, the use of list prices for biologics and anti-JAKs may not reflect the real-world scenario because payers may receive discounts and confidential reimbursements from manufacturers. Finally, based on the current macroeconomic conditions in Argentina, special attention should be paid to the evolution of drug prices. This study has several strengths. First, we created a budget impact model that fully accounts for Argentine health system characteristics and clinical practice. Second, epidemiology, resource utilization, cost data, and estimates were produced in collaboration with local experts. All costs of health-related events were calculated using micro-costing methods that included health resource identification, quantification, utilization rates, and unit costs, allowing for robust estimates of cost parameters at the local level. Finally, the budget impact model included a deterministic sensitivity analysis, which showed the uncertainty of the results.

In conclusion, our results could inform payers responsible for efficient budget resource allocation and other healthcare stakeholders and researchers. Future studies assessing the benefits of higher remission rates of RA compared to the costs derived from treatment with new molecules would further contribute to measuring the impact of using resources available for RA in Argentina. In the base-case analysis and in most sensitivity analyses, upadacitinib resulted in marginal incremental cost in the treatment for patients with moderate to severe RA and cDMARD-IR.
