# Supplementary material for: Budget impact of upadacitinib in patients with moderate to severe rheumatoid arthritis in Argentina
Source: Rev Peru Med Exp Salud Publica. 2024 Jun 17;41(2):129–39. doi: 10.17843/rpmesp.2024.412.12934 (PMC11300694; doi:10.17843/rpmesp.2024.412.12934)
Supplement: Supplementary material. — Available in the electronic version of the RPMESP. [file rpmesp-41-02-12934-s001.docx]

**Material suplementario**

**Tabla S1.** Frecuencia y costos unitarios de los principales recursos sanitarios asociados con el costo del monitoreo de los tratamientos, USD^a^

|  | **Pre-tratamiento (mensual)** | **Primeros 6 meses (mensual)** | **Mantenimiento (anual)** | **Costo unitario, seguridad social** | **Costo Unitario, sector privado** |
| --- | --- | --- | --- | --- | --- |
| Consulta reumatología | 0,99 | 2,49 | 3,75 | $10,83 | $14,88 |
| Hemograma completo | 0,89 | 1,75 | 3,25 | $2,91 | $3,08 |
| Eritrosedimentación | 0,89 | 2,00 | 3,25 | $0,77 | $1,02 |
| Factor reumatoide | 0,89 | - | - | $4,82 | $5,13 |
| Proteína C reactiva | 0,82 | 1,60 | 3,10 | $4,82 | $5,13 |
| Función renal | 0,89 | 1,95 | 3,25 | $6,74 | $7,19 |
| Función hepática | 0,93 | 2,00 | 3,00 | $6,27 | $7,70 |
| Perfil bioquímico | 0,89 | 1,75 | 3,00 | $5,49 | $6,17 |
| Radiografía de tórax | 0,81 | - | 0,34 | $9,35 | $11,82 |
| Serología de la hepatitis | 0,64 | - | 1,00 | $158,00 | $210,35 |
| Serología VIH | 0,53 | - | 1,00 | $8,48 | $11,28 |
| Prueba de Mantoux | 0,10 | - | 0,33 | $14,34 | $20,52 |
| Radiografía de manos y pies | 1,00 | - | 1,00 | $12,29 | $13,91 |
| Acitrulinas | 0,90 | - | - | $48,18 | $51,30 |

^a^Costos son mostrados en dólares estadounidenses 2024.

**Tabla S2.** Tasa de prevalencia de eventos adversos severos por paciente/año para cada clase de fármaco.

| Clase de fármaco | Tasa por paciente-año |
| --- | --- |
| DMARc | 0,095 |
| DMARb | 0,049 |
| Inhibidor JAK | 0,152 |

DMARc: fármaco antirreumático convencional modificador de la enfermedad, DMARb: fármaco antirreumático biológico modificador de la enfermedad

Las tasas de prevalencia de eventos adversos se extrapolaron a una clase de fármacos. Así, la tasa de EA de baricitinib se consideró la tasa de EA de todos los inhibidores de JAK, la tasa de adalimumab se aplicó a todos los biológicos y la tasa de EA del brazo placebo se aplicó a los DMARc. Aunque este enfoque representa una simplificación de los perfiles de seguridad de los tratamientos de la AR, se considera un enfoque conservador.

**Tabla S3**. Impacto presupuestario neto por categoría de costos, USD^a^

| Categoría de costo | Año 1 | Año 2 | Año 3 | Año 4 | Año 5 |
| --- | --- | --- | --- | --- | --- |
| Seguridad social |  |  |  |  |  |
| Adquisición y administración con Upa | 14.278 | 14.392 | 14.519 | 14.678 | 14.825 |
| Adquisición y administración sin Upa | 14.172 | 14.172 | 14.172 | 14.172 | 14.172 |
| Diferencia | 106 | 221 | 348 | 507 | 654 |
| Monitoreo con Upa | 3.258 | 3.258 | 3.258 | 3.258 | 3.258 |
| Monitoreo sin Upa | 3.258 | 3.258 | 3.258 | 3.258 | 3.258 |
| Diferencia | 0 | 0 | 0 | 0 | 0 |
| Evento adverso con Upa | 32 | 34 | 35 | 37 | 38 |
| Evento adverso sin Upa | 31 | 31 | 31 | 31 | 31 |
| Diferencia | 1 | 3 | 4 | 6 | 7 |
| Total Diferencia | 108 | 223 | 352 | 512 | 660 |
| Sector privado |  |  |  |  |  |
| Adquisición y administración con Upa | 14.312 | 14.423 | 14.546 | 14.701 | 14.846 |
| Adquisición y administración sin Upa | 14.209 | 14.209 | 14.209 | 14.209 | 14.209 |
| Diferencia | 103 | 214 | 337 | 492 | 637 |
| Monitoreo con Upa | 3.970 | 3.970 | 3.970 | 3.970 | 3.970 |
| Monitoreo sin Upa | 3.970 | 3.970 | 3.970 | 3.970 | 3.970 |
| Diferencia | 0 | 0 | 0 | 0 | 0 |
| Evento adverso con Upa | 46 | 48 | 50 | 52 | 53 |
| Evento adverso sin Upa | 44 | 44 | 44 | 44 | 44 |
| Diferencia | 2 | 4 | 6 | 8 | 10 |
| Total Diferencia | 105 | 218 | 343 | 500 | 646 |

^a^Costos son mostrados en dólares estadounidenses 2024.

**Tabla S4.** Impacto presupuestario neto por esquema de tratamiento y año

|  | **Diferencia de costos (con upadacitinib vs sin upadacitinib), USD^a^** | | | | | |
| --- | --- | --- | --- | --- | --- | --- |
| **Esquema de tratamiento** | **Año 1** | **Año 2** | **Año 3** | **Año 4** | **Año 5** | **Total** |
| UPA 15 mg + MTX | 881 | 1.830 | 2.911 | 4.027 | 4.916 | 881 |
| ABT IV + MTX | -79 | -163 | -260 | -364 | -449 | -79 |
| ABT SC + MTX | -58 | -121 | -193 | -277 | -347 | -58 |
| ADA + MTX | -11 | -22 | -36 | -56 | -75 | -11 |
| BRC + MTX | -36 | -75 | -119 | -167 | -192 | -36 |
| Intensivo DMARc^b^ | -9 | -20 | -33 | -57 | -81 | -9 |
| CTZ + MTX | -51 | -106 | -172 | -255 | -329 | -51 |
| ETN + MTX | -101 | -210 | -341 | -455 | -565 | -101 |
| GOL + MTX | -55 | -114 | -183 | -258 | -321 | -55 |
| IFX + MTX | -56 | -117 | -168 | -187 | -187 | -56 |
| RTX + MTX | -86 | -178 | -285 | -345 | -395 | -86 |
| SRL + MTX | -46 | -96 | -152 | -213 | -245 | -46 |
| TCZ IV + MTX | -79 | -165 | -262 | -366 | -421 | -79 |
| TCZ SC + MTX | -68 | -140 | -225 | -322 | -404 | -68 |
| TFC + MTX | -39 | -80 | -130 | -192 | -247 | -39 |
| Total | 108 | 223 | 352 | 512 | 660 | 108 |

ABT=abatacept; ADA=adalimumab; USD=dólares estadounidenses; BRC=baricitinib; DMARc=fármaco antirreumático convencional modificador de la enfermedad; CTZ=certolizumab pegol; ETN=etanercept; gol=golimumab; IFX=infliximab; IV=intravenoso; MTX=metotrexato; RTX=rituximab Ab; SC = subcutáneo; SRL = sarilumab; TCZ = tocilizumab; TFC = tofacitinib; UPA = upadacitinib.

^a^ Los costos se muestran en dólares estadounidenses de 2024.

^b^ DMARc Intensivo considera que la hidroxicloroquina es de 6,5 mg/kg/d, la prednisona de 7,5 mg/día, la sulfasalazina de 2 g/día y el metotrexato de 20 mg/semana.

**Material Suplementario**

**Cuestionario administrado a los expertos del Panel Delphi**

**
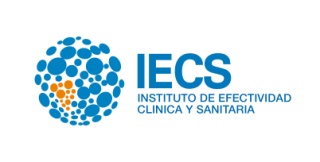
**

**MONITOREO Y TRATAMIENTO DE LA ARTRITIS REUMATOIDEA EN PACIENTES ADULTOS EN ARGENTINA**

**Ejercicio de validación y consenso de expertos**

**INICIALES SUYAS _________ FECHA: 28-06-2019**

Estimado participante, estamos encantados de contar con su participación en esta actividad. La misma tiene como objetivo relevar y/o validar datos epidemiológicos y del manejo de la Artritis Reumatoidea (AR) en Argentina.

Este proyecto de investigación es llevado a cabo, por el Instituto de Efectividad Clínica y Sanitaria de Argentina (IECS), financiado con un subsidio de investigación de la compañía farmacéutica AbbVie. Los parámetros relevados serán utilizados para la realización de un análisis de costo-efectividad. Las preguntas que usted y el resto de los expertos en el tema contestarán a continuación corresponden a información que no ha podido ser caracterizada adecuadamente en la mencionada búsqueda de literatura científica o que consideramos deben ser validados por expertos en la materia. Por lo tanto, apelamos a su conocimiento y experiencia en el tema para ayudar a dar respuesta a estas incógnitas, necesarias para llevar adelante nuestra evaluación.

Es importante remarcar que toda la información provista por usted será tratada en forma confidencial.

**Sobre las actividades y agenda**

Al inicio de la actividad se presentará el proyecto de investigación y las instrucciones sobre cómo rellenar el cuestionario. Luego habrá un período de tiempo para el llenado del cuestionario. Los cuestionarios respondidos se entregarán al equipo de IECS. A continuación, tendremos una pausa de 30 minutos, durante la cual el equipo de IECS relevará las respuestas del panel. Al retomar las actividades se devolverán los cuestionarios. A continuación, habrá un espacio de discusión de los parámetros generados, buscando arribar a un consenso, para luego proceder al cierre de la actividad.

**Enfoque de la información que solicitamos**

**Perspectiva:** Al responder sobre los tratamientos y recursos utilizados tanto durante el seguimiento habitual como en los efectos adversos, usted debe pensar en un escenario posible y promedio de Argentina, que incluya la variabilidad esperada proveniente del Sistema de salud Argentino.

**Población:** La población son pacientes adultos con AR.

**Definición del caso sobre el que realizaremos las preguntas**

El caso ejemplo sobre el cual realizaremos las preguntas se trata de un paciente adulto hipotético con diagnóstico de AR moderada o severa con respuesta inadecuada a fármacos modificadores de la enfermedad (DMARD, sigla del inglés *disease-modifying antirheumatic drugs*) convencionales y/o biológicos según se especifique. A continuación, le mostramos cuales son los criterios que, a los efectos de este cuestionario, usaremos para clasificar a los diferentes estadios de la enfermedad:

Artritis reumatoide baja actividad: DAS28≤3,2

Artritis reumatoide moderada actividad: DAS28 > 3,2 - ≤ 5,1

Artritis reumatoide severa actividad: DAS28 > 5,1

A su vez, un cambio en el DAS28 de 1,2 se considera significativo y un valor de DAS28 < 2,6 significa remisión de la enfermedad.

**Tipo de información solicitada:**

La información que le pediremos es sobre porcentajes, cantidades temporales (en días, meses, años, etc.), cantidades de recursos médicos (número de consultas, laboratorios, etc.). A su vez, en algunos casos le pediremos que responda por el orden en la secuencia de tratamientos (posición esperada de cada tratamiento disponible en Argentina), en otros por su percepción de la participación de cada tratamiento en el mercado (en términos porcentuales) y en otros sobre los tiempos esperados de tratamiento (en meses).

A su vez, le consultaremos sobre la utilización esperada de recursos médicos durante los tratamientos. En particular le preguntaremos sobre la siguiente información:

*Porcentaje del total de pacientes:* Usted debe contestar sobre el porcentaje de pacientes que espera que utilicen al menos una vez el recurso sanitario analizado. El porcentaje se refiere a cuántos pacientes de cada 100 pacientes con psoriasis en placa moderada o severa en Argentina. Este valor puede ser entre 0% a 100%. Por ejemplo, si en el seguimiento de un paciente durante el tratamiento se espera que la mitad de los mismos realice una determinación de su valor de glucemia, debería indicar que el 50% de los pacientes utiliza el recurso “determinación del valor de glucemia”.

*Cantidades del recurso (valores base, rango del intervalo de confianza al 95%):* En este caso se le solicita indicar, de acuerdo a lo que solicite la pregunta, la cantidad de unidades del recurso que espera que un paciente utilice por el tiempo indicado (por ejemplo, durante 6 meses). En todos los casos debe indicar un valor promedio y un mínimo y máximo del intervalo de confianza del 95%.

1. **Llenado adecuado del cuestionario**

Figura 1: Tipo de respuestas válidas para el llenado del cuestionario


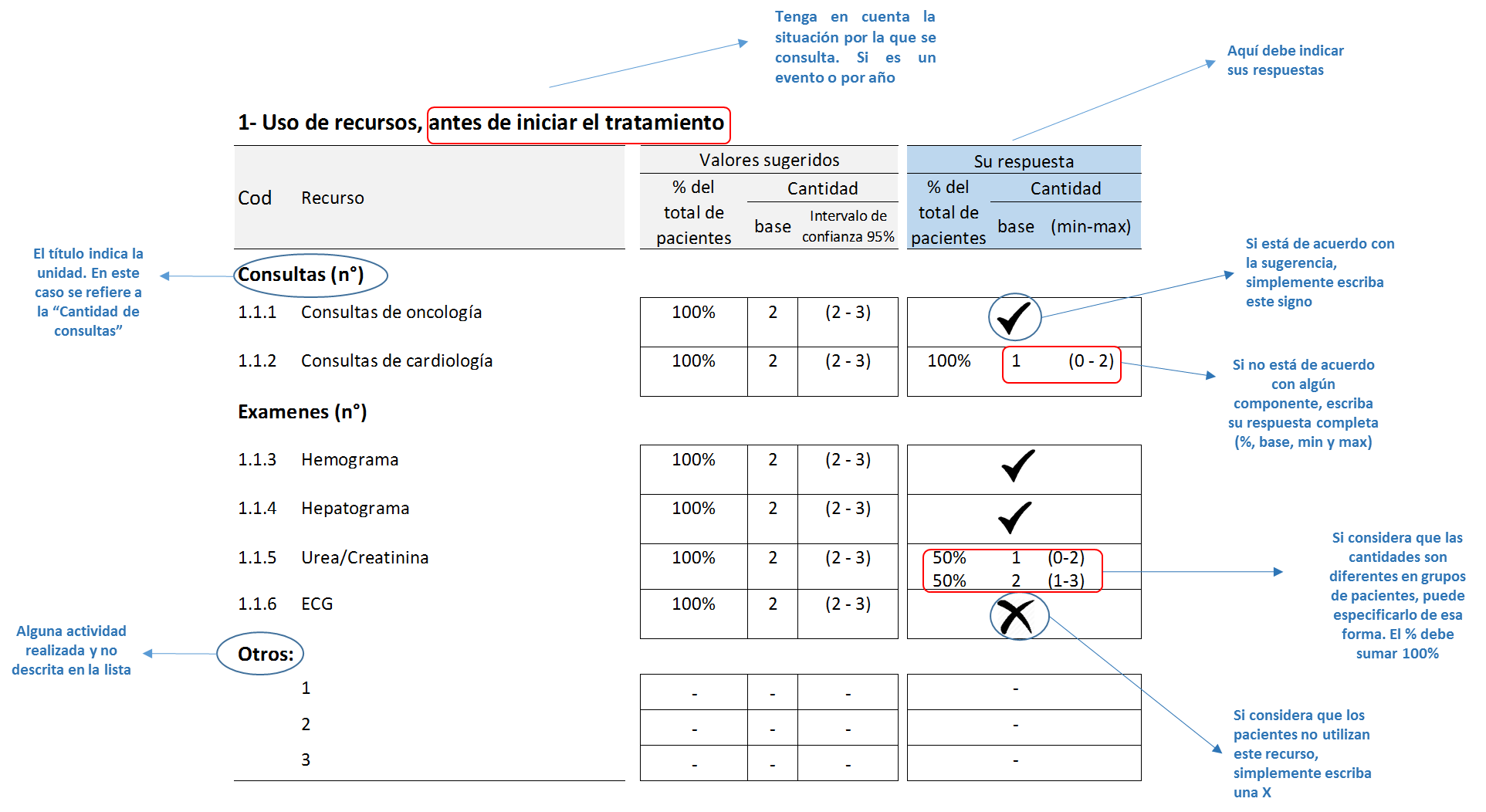


**CUESTIONARIO**

Recuerde que su respuesta no debe reflejar su experiencia y práctica, sino que debe pensar en la práctica habitual en nuestro país, desde la perspectiva del sistema de salud de Argentina.

**1- Prevalencia e incidencia de AR y distribución de los grados de severidad.**

La prevalencia se refiere al número de pacientes con diagnóstico de AR cada 1.000 habitantes y la incidencia a los nuevos casos de AR cada 100.000 habitantes por año. En base a una búsqueda bibliográfica fuentes, nos gustaría saber si considera que son apropiados los valores sugeridos o si conoce otro dato o fuente para sugerirnos, así como las distribuciones de cada grado de severidad de la enfermedad en nuestro país.

**Búsqueda bibliográfica y justificación de los valores sugeridos:** se identificaron cuatro estudios que describen la prevalencia de AR en Argentina. Peláez-Ballesta y col. publicaron en 2018 un estudio de corte transversal que describe la prevalencia de enfermedades músculo esqueléticas en cuatro poblaciones indígenas de Latinoamérica, una de ellas de Argentina.^1^ Respecto a la AR, la prevalencia encontrada fue de 1,3‰, y en un sub análisis de la comunidad Qom de Argentina la prevalencia fue del 2,4‰. Cabe aclarar que diferentes estudios han mostrado una prevalencia aumentada de enfermedades reumáticas en población indígena. Scublinsky y col. publicaron en 2010 un estudio de corte transversal que evaluó la prevalencia de AR en la ciudad de Luján utilizando una técnica de captura y re captura, tomando un registro local de pacientes con AR como fuente principal y una encuesta telefónica seguida de una evaluación por médico especialista, en caso de corresponder, como fuente secundaria; se observó una tasa de prevalencia de 9,4‰ (IC 95%: 8,6-10,2‰).^2^ Spindler y col publicaron en 2002 un estudio en el cual estimaron la prevalencia de la enfermedad en Tucumán, a partir de un registro de especialistas, encontrándose una prevalencia de 1,97‰ (IC95%: 1,8-2,0‰). ^3^ Di y col publicaron en 2016 un estudio que estimó la prevalencia e incidencia de AR en CABA a partir de diferentes bases de datos de pacientes afiliados al plan de salud de una organización de cuidados en salud. ^4^ Dado que este estudio utiliza los criterios clasificatorios 2010 para AR, que también incorporó la metodología de captura y re captura, y representa a una amplia población de nuestro país, por lo que sugieren los valores de este último como los más representativos (ver Tabla 1).

Respecto a la incidencia, los valores sugeridos provienen del único estudio local relevado en la búsqueda bibliográfica.

En cuanto a la distribución según severidad, los porcentajes sugeridos provienen de la opinión de siete expertos, para un documento reunidos por el Instituto de Efectividad Clínica Sanitaria (IECS), en 2017.

Tabla 1. Parámetros epidemiológicos de artritis reumatoidea en Argentina.

| Prevalencia, incidencia, severidad | Valores sugeridos | Su respuesta |
| --- | --- | --- |
|  | Base IC 95% | Base IC 95% |
| **Prevalencia de AR en Argentina** | | |
| Según Di y col (2016) | 3,24 2,98-3,59 |  |
| **Incidencia de AR en Argentina** | | |
| Según Di y col | 18,50 16,70-20,40 |  |
| **Distribución de los casos según severidad** | | |
| Leve | 49% |  |
| Moderada | 22% |  |
| Severa | 29% |  |
| Total | 100 | 100 |

**2- Esquemas de tratamiento en la Argentina**

2.1 ¿Qué porcentaje de pacientes con **AR moderada o severa** en Argentina estima usted que presentan respuesta inadecuada a DMARD convencionales (RI-cDMARD)?

Arturi y col publicaron en el 2008 un estudio prospectivo realizado en un centro de referencia de la especialidad en CABA, en el que se observó que, de 182 pacientes con AR, 44,5% presentaban indicación de recibir terapia biológica según guías de práctica clínica y 44% por opinión del médico evaluador.^5^ Estos resultados son concordantes con la bibliografía internacional.

Tabla 2. Porcentaje de pacientes con AR moderada/severa con respuesta inadecuada a DMARD convencionales (RI-cDMARD)

|  | **Valor sugerido** | **Su respuesta** |
| --- | --- | --- |
| **% pacientes candidatos a tratamiento de segunda línea** (**RI-cDMARD),** | 44,5% |  |

2.2 Para usted, ¿qué porcentaje de pacientes con **AR moderada o severa**, en Argentina, presenta respuesta inadecuada al tratamiento con un primer agente biológico (RI-bDMARD)? ¿Y a un segundo agente biológico?

Rolón Campuzano y col publicaron en 2018 un estudio retrospectivo, llevado a cabo en cinco centros de Argentina se reportó que el 33% y el 35,9% de los pacientes suspendieron el primer y el segundo agente biológico, respectivamente, por ineficacia. ^6^

Tabla 3 Porcentaje de pacientes con respuesta inadecuada a DMARD biológicos (RI-bDMARD)

| **Respuesta inadecuada a agentes biológicos (RI-bDMARD)** | **Valor sugerido** | **Su respuesta** |
| --- | --- | --- |
| Suspensión de 1° agente biológico | 33% |  |
| Suspensión de 2° agente biológico | 35,9% |  |

**3. Distribución de los tratamientos en Argentina**

3.1 Para usted, ¿en qué porcentaje (%) se utilizan, en Argentina, los siguientes tratamientos para la AR moderada a severa co**n respuesta inadecuada a DMARD convencionales.** (pacientes RI-cDMARD**)**? Por favor considere los porcentajes teniendo en cuenta que la totalidad debe sumar 100% y recuerde que los resultados pretenden reflejar la realidad de **todo el país**.

**Justificación:** los porcentajes propuestos se basan en los resultados del estudio de Rolón Campuzano y col publicado en 2018, llevado a cabo en cinco centros de Argentina y en opinión de expertos recabada en 2017. Dichos porcentajes presentan pequeñas modificaciones para poder incorporar drogas y estrategias de tratamiento que no se encontraban incluidas en estos estudios: combinación de DMARD convencionales, sarilumab, baricitinib y “biosimilares” de etanercept, infliximab y rituximab.

Respecto a la terapia combinada con DMARD convencionales, si bien la actividad moderada y severa son factores de mal pronóstico, lo cual favorecería la indicación de un agente biológico por sobre la terapia combinada con DMARD convencionales (guía EULAR) en la práctica diaria, por diversos motivos, se contempla la indicación de terapia DMARD convencionales combinada. En consecuencia, se incluyó dentro de las alternativas, proponiéndose un porcentaje estimativo.

**Aclaración:** ante la ausencia de respaldo bibliográfico, se proponen los mismos porcentajes para AR **moderada** y **severa**. ¿Está de acuerdo?

SI NO

Si no está de acuerdo, justifique:

Tabla 4. Distribución de tratamientos recibidos por pacientes con AR moderada/severa RI-cDMARD en Argentina

| **Tratamientos de segunda línea (pacientes con falla a DMARD convencionales)** | **Valores sugeridos (%)** | **Su respuesta** |
| --- | --- | --- |
| Combinación de DMAR convencionales | 24 |  |
| Etanercept | 24 |  |
| Adalimumab | 15 |  |
| Infliximab | 2 |  |
| Certolizumab | 6 |  |
| Golimumab | 3 |  |
| Abatacept subcutáneo | 4 |  |
| Abatacept endovenoso | 2 |  |
| Tocilizumab subcutáneo | 3 |  |
| Tocilizumab endovenoso | 1 |  |
| Rituximab | 4 |  |
| Sarilumab | 2 |  |
| Tofacitinib | 5 |  |
| Baricitinib | 2 |  |
| Biosimiliar etanercept | 1 |  |
| Biosimilar infliximab | 1 |  |
| Biosimilar rituximab | 1 |  |
| Total | 100 | **100** |

3.2 Para usted, ¿en qué porcentaje (%) se utilizan, en Argentina, los siguientes tratamientos para la AR moderada a severa con respuesta inadecuada a agentes biológicos (RI-bDMARD)? Por favor considere los porcentajes teniendo en cuenta que la totalidad debe sumar 100% y recuerde que los resultados pretenden reflejar la realidad de **todo el país**.

**Justificación:** los porcentajes propuestos se basan en los resultados del estudio de Rolón-Campuzano y col publicado en 2018, llevado a cabo en cinco centros de Argentina, y en opinión de expertos recabada en 2017. Dichos porcentajes presentan pequeñas modificaciones para poder incorporar drogas que no se encontraban incluidas en estos estudios: sarilumab, baricitinib y “biosimilares” de etanercept, infliximab y rituximab.

**Aclaración:** ante la ausencia de respaldo bibliográfico, se proponen los mismos porcentajes para AR **moderada** y para AR **severa**. ¿Está de acuerdo?

SI NO

Si no está de acuerdo, justifique:

Tabla 5 Distribución de tratamientos recibidos por pacientes con AR moderada/severa RI-bDMARD en Argentina

| **Tratamientos de tercera línea (pacientes con falla a agentes biológicos)** | **Valores sugeridos** | **Su respuesta** |
| --- | --- | --- |
| Etanercept | 17 |  |
| Adalimumab | 13 |  |
| Infliximab | 1 |  |
| Certolizumab | 4 |  |
| Golimumab | 1 |  |
| Abatacept subcutáneo | 15 |  |
| Abatacept endovenoso | 5 |  |
| Tocilizumab subcutáneo | 13 |  |
| Tocilizumab endovenoso | 5 |  |
| Rituximab | 7 |  |
| Sarilumab | 2 |  |
| Tofacitinib | 10 |  |
| Baricitinib | 2 |  |
| Biosimiliar etanercept | 1 |  |
| Biosimilar infliximab | 1 |  |
| Biosimiliar rituximab | 3 |  |
| Total | 100 | 100 |

**4. Secuencias de tratamiento**

En el modelo se consideran secuencias de hasta cinco tratamientos. Aunque estas secuencias de tratamiento podrían ser una simplificación excesiva de las verdaderas líneas de tratamiento, esto está en línea con lo realizado en evaluaciones previas presentadas a instituciones de referencia y en publicaciones internacionales. ^7-10^

Upadacitinib se compara con las alternativas incluidas como primera línea de tratamiento de cada secuencia. A los fines del modelo, la asunción es que los tratamientos posteriores son los mismos para los diferentes tratamientos de primera línea, excepto en situaciones específicas (por ejemplo, cuando se evaluaron los tratamientos posteriores seleccionados en la primera línea). Esto es para evitar que los resultados sean determinados por diferencias en tratamientos posteriores y no por diferencias en la costo-efectividad de los tratamientos evaluados.

Para la población de AR moderada activa con respuesta inadecuada a DMARD convencionales (RI-cDMARD), el modelo permite la transición de pacientes con enfermedad moderada activa a enfermedad severa activa en función de la trayectoria de las puntuaciones DAS28. Una vez que los pacientes pasan al estado de la enfermedad severa activa, se convierten en elegibles para los productos biológicos.

Las secuencias de tratamiento específicas modeladas en cada población objetivo se describen de la Tabla 6 a la Tabla 9**. De considerar que existen estrategias o secuencias de tratamientos importantes no consideradas, agregue las dos más importantes en los renglones destinados a sugerencias. De considerar que alguna de las secuencias no es válida en nuestro país, márquelo en la columna correspondiente.**

Tabla 6 Secuencias de tratamiento consideradas en población de AR de actividad moderada RI-cDMARD

| **Secuencia** | **Tratamiento 1** | **Tratamiento 2** | **Tratamiento 3** | **¿Secuencia Válida?** |
| --- | --- | --- | --- | --- |
| 1 | UPA 15mg + MTX | Mtx | Pal |  |
| 2 | Mtx | Pal | N/A |  |
| 3 | UPA 15mg | Pal | N/A |  |
| 4 | cDMARD intensivo | Pal | N/A |  |
| Sugerencia 1 |  |  |  |  |
| Sugerencia 2 |  |  |  |  |

**Abreviaturas:** ADA-adalimumab; cDMARD-medicamento antirreumático modificador de la enfermedad convencional; RI: Respuesta inadecuada; MTX-metotrexato;N/A: No aplicable; Pal-Cuidados paliativos; UPA-upadacitinib.

Tabla 7 Secuencias de tratamiento consideradas en población con AR moderada con RI- cDMARD activa después de la transición a la AR

| **Secuencia** | **Tratamiento 1** | **Tratamiento 2** | **Tratamiento 3** | **Tratamiento 4** | **¿Secuencia válida?** |
| --- | --- | --- | --- | --- | --- |
| 1 | ADA + MTX | RTX + MTX | TCZ IV + MTX | Mtx |  |
| 2 | Ada | Etn | Pal | Pal |  |
| Sugerencia 1 |  |  |  |  |  |
| Sugerencia 2 |  |  |  |  |  |

**Abreviaturas:** ADA-adalimumab; cDMARD-medicamento antirreumático modificador de la enfermedad convencional; RI: Respuesta inadecuada; MTX-metotrexato;N/A: No aplicable; Pal-Cuidados paliativos; UPA-upadacitinib.

Tabla 8 Secuencias de tratamiento consideradas en población AR severa RI- cDMARD

| **Secuencia** | **Tratamiento 1** | **Tratamiento 2** | **Tratamiento 3** | **Tratamiento 4** | **Tratamiento 5** | **¿Secuencia válida?** |
| --- | --- | --- | --- | --- | --- | --- |
| 1 | UPA 15mg + MTX | RTX + MTX | TCZ IV + MTX | Mtx | Pal |  |
| 3 | ABT SC + MTX | RTX + MTX | TCZ IV + MTX | Mtx | Pal |  |
| 4 | ABT IV + MTX | RTX + MTX | TCZ IV + MTX | Mtx | Pal |  |
| 5 | ADA + MTX | RTX + MTX | TCZ IV + MTX | Mtx | Pal |  |
| 6 | BRC + MTX | RTX + MTX | TCZ IV + MTX | Mtx | Pal |  |
| 7 | CTZ + MTX | RTX + MTX | TCZ IV + MTX | Mtx | Pal |  |
| 8 | ETN + MTX | RTX + MTX | TCZ IV + MTX | Mtx | Pal |  |
| 9 | GOL + MTX | RTX + MTX | TCZ IV + MTX | Mtx | Pal |  |
| 10 | IFX + MTX | RTX + MTX | TCZ IV + MTX | Mtx | Pal |  |
| 11 | SRL + MTX | RTX + MTX | TCZ IV + MTX | Mtx | Pal |  |
| 12 | TCZ IV + MTX | RTX + MTX | ADA+MTX | Mtx | Pal |  |
| 13 | TCZ SC + MTX | RTX + MTX | ADA+MTX | Mtx | Pal |  |
| 14 | TFC + MTX | RTX + MTX | TCZ IV + MTX | Mtx | Pal |  |
| 15 | UPA 15mg | Etn | Pal | N/A | N/A |  |
| 16 | Ada | Etn | Pal | N/A | N/A |  |
| 17 | Brc | Etn | Pal | N/A | N/A |  |
| 18 | CTZ | Etn | Pal | N/A | N/A |  |
| 19 | Etn | Ada | Pal | N/A | N/A |  |
| 20 | Srl | Etn | Pal | N/A | N/A |  |
| 21 | TCZ IV | Etn | Pal | N/A | N/A |  |
| 22 | TCZ SC | Etn | Pal | N/A | N/A |  |
| 23 | Tfc | Etn | Pal | N/A | N/A |  |
| Sugerencia 1 |  |  |  |  |  |  |
| Sugerencia 2 |  |  |  |  |  |  |

**Abreviaturas:** ABT-abatacept; ADA-adalimumab; BRC-baricitinib; cDMARD-medicamento antirreumático modificador de la enfermedad convencional; CTZ-certolizumab pegol; ETN-etanercept; GOL-golimumab; INF-infliximab: RI-Respuesta inadecuada; IV-Infusión intravenosa; MTX-metotrexato; N/A-No aplicable; Pal-cuidados paliativos; AR-Artritis reumatoidea; RTX-rituximab; SC-Inyección subcutánea; SRL-sarilumab; TCZ-tocilizumab; TFC-tofacitinib; UPA-upadacitinib.

Tabla 9. Secuencias de tratamiento consideradas en población con AR severa RI-bDMARD, elegible y no elegible para RTX

| **Secuencia** | **Tratamiento 1** | **Tratamiento 2** | **Tratamiento 3** | **Tratamiento 4** | **¿Secuencia válida?** |
| --- | --- | --- | --- | --- | --- |
| 1 | UPA 15mg + MTX | TCZ IV + MTX | Mtx | Pal |  |
| 2 | ABT IV + MTX | TCZ IV + MTX | Mtx | Pal |  |
| 3 | BRC + MTX | TCZ IV + MTX | Mtx | Pal |  |
| 4 | GOL + MTX | TCZ IV + MTX | Mtx | Pal |  |
| 5 | SRL + MTX | TCZ IV + MTX | Mtx | Pal |  |
| 6 | RTX + MTX | TCZ IV + MTX | Mtx | Pal |  |
| 7 | TCZ IV + MTX | Mtx | Pal | N/A |  |
| 8 | TCZ SC + MTX | Mtx | Pal | N/A |  |
| Sugerencia 1 |  |  |  |  |  |
| Sugerencia 2 |  |  |  |  |  |

**Abreviaturas:** ABT-abatacept; bDMARD fármaco antirreumático modificador de la enfermedad biológico; BRC-baricitinib; GOL-golimumab; RI: Respuesta inadecuada; IV-Infusión intravenosa; MTX-metotrexato; N/A-No aplicable; Pal-cuidados paliativos; SC-Inyección subcutánea; SRL-sarilumab; TCZ-tocilizumab; UPA-upadacitinib.

**5. Estimación de incorporación de upadacitinib a la práctica:**

De cada 100 pacientes con diagnóstico de AR moderada-severa con respuesta inadecuada a DMARD convencionales o respuesta inadecuada a DMARD biológicos, ¿cuántos estima que recibirán upadacitinib en el primer año, en caso de comenzar a estar disponible en nuestro país? ¿Considera que este valor se incrementará, se mantendrá o se reducirá en los años subsiguientes? Refleje estas consideraciones con valores aproximados en las tablas que siguen.

Tabla 10. Utilización de upadacitinib en el tratamiento de pacientes con AR moderada/severaRI-cDMARD

| **Tratamiento** | **% Pac AR moderada/severa Año 1** | **% Pac AR moderada/severa Año 2** | **% Pac AR moderada/severa Año3** | **% Pac AR moderada/severa Año 4** | **% Pac AR moderada/severa Año 5** |
| --- | --- | --- | --- | --- | --- |
| UPA 15mg |  |  |  |  |  |

Tabla 11 Utilización de upadacitinib en el tratamiento de pacientes con AR moderada/severa RI-bDMARD

| **Tratamiento 1** | **% Pac AR moderada/severa Año 1** | **% Pac AR moderada/severa Año 2** | **% Pac AR moderada/severa Año3** | **% Pac AR moderada/severa Año 4** | **% Pac AR moderada/severa Año 5** |
| --- | --- | --- | --- | --- | --- |
| UPA 15mg |  |  |  |  |  |

**6. Estimación de desplazamiento de otros tratamientos**

En el caso de haber indicado que considera que el upadacitinib se utilizará al menos en alguna proporción de pacientes. Indique a continuación qué alternativas actualmente en uso serán desplazadas por esta incorporación. Recuerde que en la pregunta 3 indicó los tratamientos utilizados en estas poblaciones.

Tabla 12. Alternativas desplazadas por upadacitinib en pacientes con AR moderada/severa RI-cDMARD

|  | **Fármaco** |
| --- | --- |
| 1 |  |
| 2 |  |

Tabla 13 Alternativas desplazadas por upadacitinib en pacientes con AR moderada/severa RI-bDMARD

|  | **Fármaco** |
| --- | --- |
| 1 |  |
| 2 |  |

**6. Utilización de recursos en el monitoreo**

Durante el tratamiento de la AR, se realizan una serie de controles a diferentes intervalos, y exámenes complementarios. Nos gustaría saber su opinión sobre la cantidad de cada uno de ellos, y el porcentaje de los pacientes a los que se les solicita esa práctica o consulta durante el seguimiento en Argentina. En la tabla se consideran diferentes momentos terapéuticos.

Nota: la fuente de las sugerencias o valores que se consignan a continuación surge de un panel Delphi conformado por siete expertos para un informe encabezado por el IECS, en 2017. Sin embargo, su opinión puede o no coincidir con estos valores.

| **Pacientes con AR MODERADO** | |  | Valores sugeridos | |  | Su respuesta | |
| --- | --- | --- | --- | --- | --- | --- | --- |
|  |  |  | % del  total de pacientes | Cantidad |  | % del  total de pacientes | Cantidad |
|  |  |  |  |  |  |  |  |
| **Al inicio del tratamiento** | |  |  |  |  |  |  |
|  | Consulta reumatólogo |  | 96% | 1 |  |  |  |
|  | Hemograma |  | 100% | 1 |  |  |  |
|  | Eritrosedimentacion |  | 100% | 1 |  |  |  |
|  | Factor Reumatoideo |  | 100% | 1 |  |  |  |
|  | Proteina C reactiva |  | 96% | 1 |  |  |  |
|  | Funcion renal |  | 100% | 1 |  |  |  |
|  | Funcion hepática |  | 100% | 1 |  |  |  |
|  | Perfil bioquímico |  | 100% | 1 |  |  |  |
|  | Radiografía de tórax |  | 94% | 1 |  |  |  |
|  | Serología Hepatitis C |  | 96% | 1 |  |  |  |
|  | Serología Hepatitis B |  | 96% | 1 |  |  |  |
|  | Serología HIV |  | 91% | 1 |  |  |  |
|  | Test de Mantoux |  | 100% | 1 |  |  |  |
|  | Otro 1: |  | - | - |  |  |  |
|  | Otro 2: |  | - | - |  |  |  |
|  | Otro 3: |  | - | - |  |  |  |
|  |  |  |  |  |  |  |  |
| **Primeros 6 meses, durante el tratamiento** | |  |  |  |  |  |  |
|  | Consulta reumatólogo |  | 100% | 2 |  |  |  |
|  | Hemograma |  | 100% | 2 |  |  |  |
|  | Eritrosedimentacion |  | 100% | 2 |  |  |  |
|  | Proteina C reactiva |  | 96% | 2 |  |  |  |
|  | Funcion renal |  | 100% | 2 |  |  |  |
|  | Funcion hepática |  | 100% | 2 |  |  |  |
|  | Perfil bioquímico |  | 100% | 2 |  |  |  |
|  | Otro 1: |  | - | - |  |  |  |
|  | Otro 2: |  | - | - |  |  |  |
|  | Otro 3: |  | - | - |  |  |  |
|  |  |  |  |  |  |  |  |
| **Por año durante la fase de mantenimiento** | |  |  |  |  |  |  |
|  | Consulta reumatólogo |  | 100% | 3 |  |  |  |
|  | Hemograma |  | 100% | 3 |  |  |  |
|  | Eritrosedimentacion |  | 100% | 3 |  |  |  |
|  | Proteina C reactiva |  | 96% | 3 |  |  |  |
|  | Funcion renal |  | 100% | 3 |  |  |  |
|  | Funcion hepatica |  | 100% | 3 |  |  |  |
|  | Perfil bioquimico |  | 100% | 3 |  |  |  |
|  | Radiografía de tórax |  | 93% | 1 |  |  |  |
|  | Test de Mantoux |  | 100% | 1 |  |  |  |
|  | Otro 1: |  | - | - |  |  |  |
|  | Otro 2: |  | - | - |  |  |  |
|  | Otro 3: |  | - | - |  |  |  |
|  |  |  |  |  |  |  |  |

| **Pacientes con AR SEVERA** | |  | Valores sugeridos | |  | Su respuesta | |
| --- | --- | --- | --- | --- | --- | --- | --- |
|  |  |  | % del  total de pacientes | Cantidad |  | % del  total de pacientes | Cantidad |
|  |  |  |  | base |  |  | base |
| **Al inicio del tratamiento** | |  |  |  |  |  |  |
|  | Consulta reumatólogo |  | 100% | 1 |  |  |  |
|  | Hemograma |  | 100% | 1 |  |  |  |
|  | Eritrosedimentacion |  | 100% | 1 |  |  |  |
|  | Factor Reumatoideo |  | 100% | 1 |  |  |  |
|  | Proteina C reactiva |  | 96% | 1 |  |  |  |
|  | Funcion renal |  | 100% | 1 |  |  |  |
|  | Funcion hepatica |  | 100% | 1 |  |  |  |
|  | Perfil bioquimico |  | 100% | 1 |  |  |  |
|  | Radiografía de tórax |  | 94% | 1 |  |  |  |
|  | Serología Hepatitis C |  | 96% | 1 |  |  |  |
|  | Serología Hepatitis B |  | 96% | 1 |  |  |  |
|  | Serología HIV |  | 94% | 1 |  |  |  |
|  | Test de Mantoux |  | 100% | 1 |  |  |  |
|  | Otro 1: |  | - | - |  |  |  |
|  | Otro 2: |  | - | - |  |  |  |
|  | Otro 3: |  | - | - |  |  |  |
|  |  |  |  |  |  |  |  |
| **Primeros 6 meses, durante el tratamiento** | |  |  |  |  |  |  |
|  | Consulta reumatólogo |  | 100% | 2 |  |  |  |
|  | Hemograma |  | 100% | 2 |  |  |  |
|  | Eritrosedimentacion |  | 100% | 2 |  |  |  |
|  | Proteina C reactiva |  | 100% | 2 |  |  |  |
|  | Funcion renal |  | 100% | 2 |  |  |  |
|  | Funcion hepatica |  | 100% | 2 |  |  |  |
|  | Perfil bioquimico |  | 100% | 2 |  |  |  |
|  | Otro 1: |  | - | - |  |  |  |
|  | Otro 2: |  | - | - |  |  |  |
|  | Otro 3: |  | - | - |  |  |  |
|  |  |  |  |  |  |  |  |
| **Por año durante la fase de mantenimiento** | |  |  |  |  |  |  |
|  | Consulta reumatólogo |  | 100% | 3 |  |  |  |
|  | Hemograma |  | 100% | 3 |  |  |  |
|  | Eritrosedimentacion |  | 100% | 3 |  |  |  |
|  | Proteina C reactiva |  | 100% | 3 |  |  |  |
|  | Funcion renal |  | 100% | 3 |  |  |  |
|  | Funcion hepatica |  | 100% | 3 |  |  |  |
|  | Perfil bioquimico |  | 100% | 3 |  |  |  |
|  | Radiografía de tórax |  | 100% | 1 |  |  |  |
|  | Test de Mantoux |  | 100% | 1 |  |  |  |
|  | Otro 1: |  | - | - |  |  |  |
|  | Otro 2: |  | - | - |  |  |  |
|  | Otro 3: |  | - | - |  |  |  |
|  |  |  |  |  |  |  |  |

**REFERENCIAS**

1. Pelaez-Ballestas I, Granados Y, Quintana R, et al. Epidemiology and socioeconomic impact of the rheumatic diseases on indigenous people: an invisible syndemic public health problem. *Ann Rheum Dis.* 2018;77(10):1397-1404.

2. Scublinsky D, Venarotti H, Citera G, et al. The prevalence of rheumatoid arthritis in Argentina: a capture-recapture study in a city of Buenos Aires province. *JCR: Journal of Clinical Rheumatology.* 2010;16(7):317-321.

3. Spindler A, Bellomio V, Berman A, et al. Prevalence of rheumatoid arthritis in Tucuman, Argentina. *The Journal of rheumatology.* 2002;29(6):1166-1170.

4. Di WT, Vergara F, Bertiller E, et al. Incidence and prevalence of rheumatoid arthritis in a health management organization in Argentina: a 15-year study. *The Journal of rheumatology.* 2016;43(7):1306-1311.

5. Arturi P, Andrea D, Citera G, Cocco JAM. Indicación de terapia biológica en pacientes con enfermedades reumáticas de la consulta ambulatoria. *Revista Argentina de Reumatología.* 2008;19(1):35.

6. Campuzano RR, Dal Pra F, Schneeberger E, et al. Patrones de tratamiento con agentes biológicos. Eficacia y sobrevida a largo plazo en pacientes con artritis reumatoidea. *REvISTA ARgENTINA DE REuMAToLogÍA.* 2018;29(4):14-19.

7. National Institute for Health and Care Excellence (NICE). Single Technology Appraisal. Baricitinib for Treating Moderate to Severe Rheumatoid Arthritis [ID979]: Committee Papers. 2017.

8. National Institute for Health and Care Excellence (NICE). Single Technology Appraisal. Tofacitinib for treatment moderate to severe active rheumatoid arthritis after the failure of disease-modifying anti-rheumatic drugs [ID526]: Committee Papers. 2017.

9. National Institute for Health and Care Excellence (NICE). Single Technology Appraisal. Sarilumab for previously treated moderate to severe active rheumatoid arthritis [ID994]: Committee Papers. 2017.

10. ScHARR UoS. Technology Assessment Report Commissioned by the NIHR HTA Programme on behalf of the National Institute for Health and Clinical Excellence: Adalimumab, etanercept, infliximab, certolizumab pegol, golimumab, tocilizumab and abatacept for the treatment of rheumatoid arthritis not previously treated with disease-modifying anti-rheumatic drugs and after the failure of conventional disease-modifying anti-rhuematic drugs only: systematic review and economic evaluation. 2013.
