# Supplementary material for: Budget impact of upadacitinib in patients with moderate to severe rheumatoid arthritis in Argentina
Source: Rev Peru Med Exp Salud Publica. 2024 Jun 17;41(2):129–39. doi: 10.17843/rpmesp.2024.412.12934 (PMC11300694; doi:10.17843/rpmesp.2024.412.12934)
Supplement: Translation

# Translation: Impacto presupuestario de upadacitinib en pacientes con artritis reumatoide moderada a severa en Argentina

**Authors:** Natalia Espinola, Anastasia Secco, Dario Balan, Diego Kanevsky, Guido Calvi, Pierre Morisset, Ariel Bardach, Federico Augustovski

**Affiliations:** 1 Instituto de Efectividad Clínica y Sanitaria, Ciudad de Buenos Aires, Argentina.; 2 AbbVie Argentina, Buenos Aires, Argentina.

**Keywords:** Impacto presupuestario, Artritis reumatoide, Argentina, upadacitinib

## Abstract

**Objetivos.:**

Analizar el impacto presupuestario de upadacitinib (UPA) 15 mg + metotrexato (MTX) para el tratamiento de la artritis reumatoide (AR) moderada a grave en pacientes con respuesta inadecuada a los fármacos antirreumáticos modificadores de la enfermedad convencionales (RI-DMARc) desde la perspectiva de la seguridad social y los sectores de salud privados en Argentina.

**Materiales y métodos.:**

Se desarrolló un modelo de análisis de impacto presupuestario para una cohorte hipotética de 100,000 adultos con cobertura de seguro de salud que fueron diagnosticados con AR en un horizonte de tiempo de 5 años. Los parámetros del modelo se obtuvieron a través de una revisión de la literatura y se validaron con expertos locales. Los costos se expresan en dólares estadounidenses (USD) para el 2024.

**Resultados.:**

La introducción de UPA 15 mg + MTX para el tratamiento de AR moderada a grave y RI-DMARc resultó en un costo incremental mínimo, con un costo acumulado total a cinco años de USD 1.855 para la seguridad social y USD 1.812 para el sector privado de salud, lo que representa aproximadamente el 2% del presupuesto total. El costo de adquisición de UPA fue la variable más influyente en el análisis de sensibilidad.

**Conclusiones.:**

La introducción de UPA 15 mg + MTX para el tratamiento de AR genera un incremento de costos marginal para el sistema de salud en Argentina, lo cual es especialmente importante en contextos de presupuestos limitados. Proporcionar estimaciones basadas en la evidencia es una herramienta valiosa para ayudar a tomar decisiones informadas sobre la asignación eficiente de recursos en salud.

## INTRODUCCIÓN

La artritis reumatoide (AR) es una enfermedad reumática autoinmune crónica de etiología desconocida que se caracteriza por una inflamación poliarticular y simétrica de las articulaciones pequeñas y grandes con posible afectación sistémica. La AR es progresiva y puede provocar la destrucción de las articulaciones, la discapacidad y un mayor riesgo de mortalidad ^1^. En Argentina, su prevalencia estimada es del 0,94% (IC 95%: 0,86% - 1,02%) y la tasa de incidencia anual es de 18,50 (IC 95%: 16,70-20,40) por cada 100.000 personas; 25,20 (22,40 - 28,00) en mujeres y 8,80 (6,80 - 10,80) en hombres ^2^^-^^4^. La AR no tiene cura; el objetivo del tratamiento es controlar la sinovitis, prevenir el daño articular, reducir las comorbilidades y mantener la función y la calidad de vida ^5^^-^^7^. La estrategia de tratamiento depende de la actividad de la enfermedad, la respuesta a los tratamientos anteriores, las comorbilidades, las preferencias de los pacientes y los aspectos relacionados con el acceso y costos, y se basa en el uso de fármacos antirreumáticos modificadores de la enfermedad (DMAR), principalmente los DMAR convencionales (DMARc). El metotrexato (MTX) es el CDMAR más utilizado. En pacientes con una respuesta inadecuada al tratamiento inicial, se administran los DMAR biológicos (DMARb), incluidos los agentes anti-factor de necrosis tumoral (anti-TNF), el abatacept, el tocilizumab, el sarilumab y el rituximab. Los DMARb requieren administración parenteral, ya sea por vía subcutánea o intravenosa. Los inhibidores de la quinasa Janus o anti-JAK son una nueva clase de fármacos introducidos recientemente, que incluyen el baricitinib, el tofacitinib y el upadacitinib; se pueden administrar por vía oral y tienen una vida media más corta que los DMARb ^6^^-^^8^.

En Argentina, el uso de CDMAR, más comúnmente MTX, como terapia de primera línea es una práctica estándar ^5^^-^^9^. El upadacitinib, un fármaco anti-JAK, ha demostrado su eficacia y seguridad en pacientes con RI-CDMAR y respuesta inadecuada a las drogas modificadoras de la enfermedad biológicas (RI-DMARb); en Argentina, se aprobó en marzo de 2020 ^10^^-^^14^. Como tratamiento de un solo fármaco, el upadacitinib también mostró su superioridad en comparación con el MTX en pacientes sin tratamiento previo con MTX con AR en etapa temprana, en pacientes con una respuesta inadecuada a MTX, y en comparación con el adalimumab y el abatacept ^14^^-^^17^. Las estrategias de tratamiento para los pacientes con artritis reumatoide moderada a severa y RI-DMARc se asocian a costos elevados. En Argentina, el costo total anual promedio de la AR sin productos biológicos fue de 3.093 USD en 2002. Las hospitalizaciones representaron el 73% de los costos médicos directos totales, mientras que los medicamentos y los procedimientos ambulatorios representaron el 16% y el 8% de los costos médicos directos totales, respectivamente ^18^.

En las últimas décadas, los sistemas de salud han experimentado un aumento creciente del gasto, lo que ha llevado a que los entornos con recursos limitados, como Argentina, requieran herramientas para la asignación eficiente y la priorización de los recursos de atención médica, al tiempo que mejoran los resultados de los pacientes. El Foro de Políticas de América Latina 2018 concluye que una de las dimensiones esenciales para la toma de decisiones es el análisis del impacto presupuestario, es decir, es esencial analizar la asequibilidad de la incorporación de nuevas tecnologías ^19^.

En Argentina, la expansión del uso de productos biológicos en pacientes con artritis reumatoide entre 2012 y 2022 podría suponer un ahorro neto acumulado de 495 000 millones de dólares en 10 años (tipo de cambio: 4,75 pesos argentinos (ARS) /dólares estadounidenses (USD)) ^20^. Sin embargo, no se han realizado estudios de evaluación económica del upadacitinib en Argentina. Nuestro objetivo fue evaluar el impacto presupuestario de la introducción del upadacitinib para el tratamiento de la artritis reumatoide en un horizonte temporal de 5 años desde la perspectiva de la seguridad social y el sector de la salud privado en Argentina.

**MENSAJES CLAVE**

Motivación para realizar el estudio. Conocer el impacto presupuestario del tratamiento con 15 mg de upadacitinib más metotrexato en pacientes con artritis reumatoide (AR) moderada a severa que no respondieron bien a los fármacos antirreumáticos convencionales.

Hallazgos principales. Upadacitinib más metotrexato implicaría un aumento mínimo de los costos para el sistema de salud en Argentina, lo que podría hacer que esta opción de tratamiento eficaz fuera más accesible para los pacientes con AR.

Implicancias. En países con presupuestos limitados, proporcionar estimaciones de costos basadas en la evidencia puede ayudar a los financiadores de la salud a asignar los recursos de manera eficiente y, al mismo tiempo, mejorar los resultados de los pacientes.

## MATERIALES Y MÉTODOS

### Marco analítico

Desarrollamos un modelo para el análisis del impacto presupuestario basado en la prevalencia utilizando un enfoque estático con una cohorte abierta en estado estacionario (Figura 1), siguiendo las pautas de los modelos de Mauskopf *et al* (2017) ^21^. La estructura analítica del modelo constaba de cuatro componentes principales que, combinados, dieron como resultado la estimación del impacto presupuestario para el financiador de la salud en el estado actual (sin upadacitinib) y la estrategia de incorporación del upadacitinib. Los componentes fueron 1) la estimación de la población que recibiría la tecnología; 2) la cuota de mercado de los medicamentos (estado actual y estrategia de incorporación del upadacitinib); 3) la estimación de los costos de adquisición, administración, monitoreo y eventos adversos relacionados con el tratamiento; y 4) los costos asociados con el manejo de los eventos adversos. El modelo se desarrolló siguiendo las recomendaciones de los principios de buenas prácticas de ISPOR ^22^.

**Figura 1:** Modelo analítico

Consideramos el mercado actual sin upadacitinib y un mercado hipotético con la introducción de upadacitinib en un horizonte temporal de 5 años y calculamos el impacto presupuestario derivado de su incorporación. Se usó upadacitinib 15 mg + metotrexato como fármaco de intervención. Los fármacos considerados en el escenario sin upadacitinib (comparadores) fueron abatacept (subcutáneo e intravenoso), adalimumab, baricitinib, certolizumab pegol, etanercept, golimumab, infliximab, rituximab, sarilumab, tocilizumab, tofacitinib (todos en combinación con metotrexato) y los DMARDc intensivos (incluyendo hidroxicloroquina 6,5 mg/kg/d, prednisona 7,5 mg/d, sulfasalazina 2 g/d y metotrexato 20 mg/semana). Los comparadores fueron seleccionados por el equipo de investigación en función de las opciones terapéuticas disponibles en el contexto argentino y aprobados por la Administración Nacional de Medicamentos, Alimentos y Tecnología Médica (ANMAT) para su comercialización.

La población elegible se estimó como la proporción de personas con AR moderada a severa de una cohorte teórica de 100.000 adultos y RI-DMARc, según las posibles tasas de incidencia y prevalencia en todo el país, tal como se describen en la literatura. El análisis se realizó desde las perspectivas del sector de la seguridad social y el sector de la salud privada en el sistema de salud argentino durante un horizonte temporal de 5 años ^23^. Los resultados no se ajustaron por tasa de descuento ni la inflación, basándose en las recomendaciones de las guías de evaluación económica ^23^.

Los supuestos clave del modelo fueron los siguientes: a) las tasas de incidencia y prevalencia se mantuvieron constantes durante el período de análisis; b) los casos perdidos por muerte, la interrupción del tratamiento o la progresión de la enfermedad se reflejaban en las tasas de prevalencia; c) el porcentaje de pacientes con AR de moderada a severa que reunían los requisitos para recibir tratamiento biológico se mantuvo constante en cada período de tiempo; y d) los costos de adquisición del medicamento asumieron que las cohortes incidentes y prevalentes recibieron tratamiento durante un año completo, ya que se desconoce el tiempo en el cual ambas cohortes se unen y comienzan el tratamiento.

Tras calcular la población que podría tratarse en el escenario sin upadacitinib y en el escenario con upadacitinib (multiplicando la población objetivo por la cuota de mercado de cada fármaco), se estimó el costo total del tratamiento para obtener el impacto presupuestario total de cada escenario. Para calcular el impacto presupuestario neto, el presupuesto sin upadacitinib se restó del presupuesto con upadacitinib (Figura 1). Este estudio presentó los costos diferenciales absolutos, la participación (%) en los costos anuales y, los costos diferenciales por miembro y por mes (PMPM) por sector de la salud. Para evaluar la robustez de los resultados, se realizó un análisis de sensibilidad determinístico modificando los siguientes parámetros en un ± 20%: precio de upadacitinib, precio de tofacitinib, precio de abatacept IV, porcentaje de pacientes que no responden a los fármacos convencionales, porcentaje de AR moderada a severa, cuota de mercado de upadacitinib y tasa de incidencia, utilizando los valores del IC del 95% (16,7-20,4).

### Población objetivo

Los datos epidemiológicos para estimar la población de pacientes elegibles para el tratamiento se obtuvieron de una revisión de la literatura y se validaron mediante un panel Delphi de expertos locales (en el material suplementario se presenta el cuestionario respondido por los expertos). Como resultado del proceso de consenso, se estimó que la prevalencia de la artritis reumatoide en adultos en Argentina era del 0,80%, lo que coincide estrechamente con lo reportado por un estudio de prevalencia bien diseñado realizado en Luján (Argentina) (0,94% [IC 95%: 0,86 - 1,02]) ^2^. Si bien, nuestros resultados no proporcionaron un panorama epidemiológico representativo, fueron una aproximación razonable de la prevalencia de la enfermedad en Argentina ^2^. Se consideró una tasa de incidencia anual de 18,50 por 100.000 personas adultas (IC 95%: 16,70 - 20,40), según se informó en un estudio realizado por una organización sanitaria local de referencia ^3^. Los expertos estimaron que el porcentaje de pacientes adultos con artritis reumatoide moderada a severa era del 64%, mientras que el 45% de estos pacientes tenían RI-DMARc y eran aptos para recibir tratamiento con upadacitinib ^24^. Los principales parámetros epidemiológicos del modelo se resumen en la Tabla 1.

**Tabla 1:** Principales parámetros epidemiológicos

Parámetros		Valor medio	Límite inferior	Límite superior	Fuente
Prevalencia de la AR en Argentina; casos por cada 1,000 personas		0,80	0,30	1,20	Scublinsky *et al*. ^2^ + Expertos
Incidencia de la AR en Argentina, casos por cada 100 000 personas		18,50	16,70	20,40	Di *et al*^3^
Distribución de los casos de AR por gravedad, %					
	Leve	36	-	-	Expertos
	Moderado	33	-	-	Expertos
	Severa	31	-	-	Expertos
Pacientes con AR moderada a severa RI-DMARc y aptos para recibir upadacitinib,%		45	36	54	Arturi *et al.* ^22^ + Expertos

RI-DMARc: respuesta inadecuada a los fármacos antirreumáticos modificadores de la enfermedad convencionales; AR: artritis reumatoide.

### Costos

El análisis se realizó desde las perspectivas de la seguridad social y el sector privado de la salud. En Argentina, el sistema de salud comprende tres subsistemas: seguridad social, sector de salud privado y sector de salud pública. La seguridad social y el sector privado de la salud representan en conjunto el 65% de la población argentina y casi el 54% del gasto total en salud (el 45% para la seguridad social y el 9% para el sector privado de la salud) ^25^. En este análisis solo se consideraron los costos médicos directos, incluidos los costos de la adquisición y administración de medicamentos, el monitoreo de la enfermedad y los eventos adversos.

Se utilizó un método de micro-costeo para estimar los costos de los eventos de salud. Este método implica identificar los recursos de salud, las tasas de uso y la cantidad. Se identificaron los costos unitarios de los recursos de salud para cada subsector (seguridad social y sector privado). El costo total esperado para cada evento resultó de la suma de los productos de estos tres componentes (tasas de uso, cantidades y costos unitarios). La identificación y medición de los recursos de salud (cantidades y tasas de uso) fueron el resultado de la revisión de la literatura y del panel Delphi. El valor de los costos unitarios se obtuvo de la base de costos unitarios del Instituto de Efectividad Clínica Sanitaria ^26^. Los costos se expresaron en dólares de 2024 (USD). El tipo de cambio nominal medio en enero de 2024 fue de 831,35 ARS por 1 USD ^27^.

Los costos de adquisición de medicamentos se derivaron de las listas de precios nacionales oficiales ^28^^,^^29^. Para el análisis utilizamos los precios de salida de laboratorio, teniendo en cuenta un descuento aproximado del 57% sobre el precio minorista ^30^. Las dosis y posologías de administración de cada agente biológico fueron proporcionados por los prospectos de los medicamentos, guías locales y un experto en reumatología ^5^^-^^9^. En Argentina, upadacitinib fue aprobado por la Administración Nacional de Medicamentos, Alimentos y Tecnología Médica (ANMAT; acrónimo en español) en diciembre de 2019.

La Tabla 2 resume el costo total anual de los medicamentos incluidos en los tratamientos, estimados utilizando el precio de salida de laboratorio por unidad y las unidades consumidas durante un año por un paciente. En el caso base, el costo anual de adquisición de upadacitinib fue de 47 USD por unidad de 15 mg (3,15 USD por mg).

**Tabla 2:** Dosificación, precio de salida de laboratorio y costo anual de adquisición de medicamentos por paciente ^a^

Tratamiento	Tamaño del vial, mg	Vía de administración	Dosis	Unidades/año	Precio salida laboratorio/mg, USD	Costo anual por paciente, USD
UPA	15	Oral	15 mg/día	365	3,15	17,251
ABT	250	IV	750 mg los días 0, 15, 30 y mensualmente a partir de entonces	42	2,72	28,588
ABT	125	SC	125 mg/semana	52	3,00	19,512
AHÍ	40	SC	40 mg/2 semanas	26	13,36	13,898
BRC	4	Oral	4 mg/día	365	6,91	10,083
CTZ	200	SC	400 mg los días 0, 15, 30 y mensualmente a partir de entonces	28	3,17	17,742
ETN	50	SC	50 mg/semana	52	6,40	16,633
GOL	50	SC	50 mg/mes	12	30,76	18,455
IFX	100	IV	3 mg/kg el día 1, la semana 2, la semana 6 y cada 8 semanas	16	3,92	6,275
MTX	10	Oral	20 mg/semana	104	0,06	58
CDMARD intensivo	-	-	^b^	-	-	630
RTX	500	SC	2000 mg cada 9 meses	8	4,88	19,513
SRL	200	SC	200 mg cada 2 semanas	26	2,88	14,969
TCZ	200	IV	8 mg/kg/mes	36	3,28	235,581
TCZ	162	SC	162 mg/semana	52	2,75	23,162
TFC	5	Oral	10 mg/día	730	3,35	12,220

ABT=abatacept; ADA=adalimumab; USD=dólares estadounidenses; BRC=baricitinib; cDMARD=fármaco antirreumático convencional modificador de la enfermedad; CTZ=certolizumab pegol; ETN=etanercept; gol=golimumab; IFX=infliximab; IV=intravenoso; MTX=metotrexato; RTX=rituximab Ab; SC = subcutáneo; SRL = sarilumab; TCZ = tocilizumab; TFC = tofacitinib; UPA = upadacitinib.

a Los costos se muestran en dólares estadounidenses de 2024.

b cDMARD Intensivo considera que la hidroxicloroquina es de 6,5 mg/kg/d, la prednisona de 7,5 mg/día, la sulfasalazina de 2 g/día y el metotrexato de 20 mg/semana.

Fuente: Elaboración propia. La información sobre los precios de los medicamentos se obtuvo de Kairos Web Argentina y SACIFyS Grupo Alfa Beta.

Por otro lado, el costo estimado de la administración intravenosa fue de 113 USD para el sector de la seguridad social y 190 USD para el sector privado; el costo de la administración subcutánea fue de 1,87 y 2,61 USD en el sector de la seguridad social y sector privado, respectivamente; la administración oral no tuvo ningún costo. A su vez, el costo anual del monitoreo de los pacientes antes del tratamiento se estimó en 160,28 USD para la seguridad social y 198,05 USD para el sector privado; el costo del primer período de tratamiento de 6 meses fue de 58,25 y 71,21 USD, para la seguridad social y el sector privado, respectivamente; y el costo mensual fue de 8,46 y 10,27 USD para la seguridad social y el sector privado, respectivamente. La Tabla S1 del material suplementario muestra los recursos identificados en esta categoría, su frecuencia y el costo unitario para ambos sectores. Las infecciones graves se consideraron el evento adverso (EA) más prevalente asociado al tratamiento. Las tasas de EA se derivaron de la presentación de baricitinib al NICE utilizando datos del estudio RA-BEAM. Las tasas de eventos adversos se extrapolaron a las clases de fármacos. Las tasas utilizadas en el modelo se presentan en la Tabla S2 del material suplementario. En cuanto a los costos relacionados con los EA, el costo de la neumonía se consideró representativo de todos los costos asociados con las infecciones graves y se estimó en 1,605 USD para la seguridad social y 2,267 USD para el sector privado.

### Distribución de los esquemas de tratamiento

Los parámetros relacionados con la distribución de la tasa de utilización de fármacos biológicos y anti-JAK fueron determinados por un panel de expertos, basándose en los patrones de tratamiento de la terapia biológica en pacientes con AR en un estudio realizado en 2018 en Argentina ^31^ y en una revisión de la literatura ^4^. Según las directrices internacionales, se prefirió el tratamiento con DMARDb o anti-JAK en lugar de la terapia combinada con un DMARc ^6^. Sin embargo, la terapia combinada con DMARc se usa en la práctica diaria en Argentina debido a la buena respuesta de los pacientes al tratamiento y al acceso limitado a algunos de los medicamentos. Como resultado, estos agentes se aceptaron como opción de tratamiento y se presentaron a los panelistas, quienes confirmaron esta práctica y acordaron una cuota de mercado. Lo mismo se aplicó para el escenario con upadacitinib. La Tabla 3 muestra la distribución porcentual de los esquemas de tratamiento. Suponemos que, en el escenario actual sin upadacitinib, las cuotas de mercado se mantuvieron constantes durante los cinco años.

**Tabla 3:** Distribución de los esquemas de tratamiento para los pacientes con una respuesta inadecuada a los DMARc: escenarios de casos con y sin upadacitinib

Esquema de tratamiento	Sin upadacitinib, % Años 1 a 5	Con upadacitinib, %				
		Año 1	Año 2	Año 3	Año 4	Año 5
UPA 15 mg + MTX	0	3,25	6,75	10,74	14,85	18,13
ABT IV+ MTX	1,27	1,08	0,87	0,64	0,38	0,18
ABT SC + MTX	3,81	3,62	3,41	3,17	2,90	2,66
ADA + MTX	13,71	13,51	13,31	13,06	12,7	12,37
BRC + MTX	1,02	0,82	0,62	0,38	0,13	0,00
CDMARD intensive	28,68	28,49	28,28	28,01	27,52	27,04
CTZ + MTX	8,12	7,93	7,72	7,48	7,17	6,89
ETN + MTX	22,72	22,33	21,92	21,42	20,99	20,57
GOL + MTX	2,54	2,35	2,14	1,90	1,64	1,42
IFX + MTX	1,27	0,89	0,47	0,13	0,00	0,00
RTX + MTX	3,43	3,04	2,63	2,16	1,89	1,67
SRL + MTX	1,02	0,82	0,62	0,38	0,13	0,00
TCZ IV+ MTX	1,02	0,82	0,62	0,38	0,13	0,00
TCZ SC + MTX	3,81	3,62	3,41	3,17	2,9	2,66
TFC + MTX	7,61	7,42	7,22	6,97	6,67	6,40
Total	100,00	100,00	100,00	100,00	100,00	100,00

ABT=abatacept; ADA=adalimumab; BRC=baricitinib; cDMARD=fármaco antirreumático convencional modificador de la enfermedad; CTZ=certolizumab pegol; ETN=etanercept; gol=golimumab; IFX=infliximab; IV=intravenoso; MTX=metotrexato; RTX=rituximab; SC=subcutáneo; SRL = sarilumab; TCZ = tocilizumab; TFC = tofacitinib; UPA = upadacitinib.

a Los cDMARD intensivos incluyeron hidroxicloroquina 6,5 mg/kg/d, prednisona 7,5 mg/d, sulfasalazina 2 g/d y metotrexato 20 mg/semana.

### Regla de decisión: umbral de impacto presupuestario

Nuestro estudio empleó la metodología utilizada por la Comisión Nacional para la Evaluación de Tecnologías Sanitarias y Excelencia Clínica del Ministerio de Salud (CONETEC) del país para estimar un umbral de alto impacto presupuestario. Este enfoque se basa en el estudio de Pichón-Riviere y colaboradores, que es particularmente relevante para los países que carecen de estimaciones propias ^32^. El valor de referencia del umbral de alto impacto presupuestario se estima en 0,00016 unidades de gasto sanitario (0,00008-0,0024). La estimación del umbral de alto impacto presupuestario en Argentina para 2023 se realizó utilizando el valor de referencia y la estimación del gasto total en salud. Esto último se estima utilizando datos del Producto Interno Bruto (PIB) y la población total de Argentina, y el promedio de los últimos diez años disponibles del gasto en salud como porcentaje del PIB ^33^^,^^34^. En consecuencia, se estimó que el umbral de alto impacto presupuestario es de 0,010 USD PMPM (0,005 -0,015 USD) para el sistema de salud.

## RESULTADOS

En la Tabla 4 se muestra el impacto presupuestario de la introducción de upadacitinib 15 mg + MTX durante un período de 5 años para los pacientes con AR moderada a severa y RI-DMARc desde la perspectiva de la seguridad social y el sector privado. Para una cohorte hipotética de 100.000 adultos con cobertura de salud, 23 pacientes con artritis reumatoide moderada a severa por año serían elegibles para recibir terapia biológica. Con la introducción de upadacitinib, a un precio de 3,15 USD por mg, el costo del tratamiento de la artritis reumatoide moderada a severa se incrementó ligeramente. En el caso del sector de la seguridad social, el costo total anual con upadacitinib fue de 17,568 USD en el primer año y 18.121 USD en el quinto año, comparado con 17,460 USD sin la introducción de upadacitinib. Para el sector privado, el costo total anual del tratamiento con upadacitinib fue de 18,328 USD en el primer año y 18,870 USD del quinto año, comparado con 18,223 USD sin la introducción de upadacitinib.

**Tabla 4:** Resultados del caso base: costos anuales con y sin upadacitinib, costos diferenciales absolutos, participación en el costo anual y costos diferenciales por miembro por mes (PMPM) de la seguridad social y el sector de la salud privado ^a^

		Costo anual total sin upadacitinib, USD	Costo anual total con upadacitinib, USD	Diferencia absoluta, USD	Participación en el costo anual, %	Costos diferenciales PMPM, USD
		A	B	C = (B-A)	C/A	D*
Seguridad social						
	Año 1	17,460	17,568	108	0,62	0,000090
	Año 2	17,460	17,648	223	1,28	0,000186
	Año 3	17,460	17,812	352	2,02	0,000293
	Año 4	17,460	17,972	512	2,93	0,000427
	Año 5	17,460	18,121	660	3,78	0,000550
	Total/Media	87,302	89,157	371	2,13	0,000309
Sector privado						
	Año 1	18,223	18,328	105	0,58	0,000087
	Año 2	18,223	18,441	218	1,19	0,000181
	Año 3	18,223	18,566	343	1,88	0,000286
	Año 4	18,223	18,723	500	2,74	0,000417
	Año 5	18,223	19,869	646	3,55	0,000539
	Total/Media	91,114	92,926	362	1,99	0,000302

USD = dólares de los Estados Unidos; PMPM = por miembro por mes.

a Los costos se muestran en dólares estadounidenses de 2024.

*El diferencial de costos anual por miembro y mes se obtuvo dividiendo el diferencial de costos anual (C) por 100.000, y el resultado se dividió por 12 meses.

La introducción de upadacitinib se asoció con un impacto presupuestario neto anual marginal, con un aumento promedio del 2% en un período de 5 años. El impacto presupuestario neto por miembro y mes (PMPM) fue de 0,0005 USD en el quinto año. Si un pagador de la seguridad social reembolsara el tratamiento con upadacitinib, el aumento promedio del presupuesto sería de 0,0003 USD PMPM. Los resultados para el sector de la salud privada son similares, con un aumento marginal de los costos. El análisis del impacto presupuestario por categoría de costo y terapias se presenta en las Tablas S2 y S3 del material suplementario.

Los resultados del análisis de sensibilidad determinístico para upadacitinib 15 mg + MTX en pacientes con AR de moderada a severa y RI-DMARc se muestran en la Figura 2. Se seleccionaron varios parámetros asociados con el aumento de la incertidumbre, y el precio unitario del upadacitinib mostró la mayor variabilidad. En el sector de la seguridad social, un aumento del 20% en el precio de upadacitinib se traduciría en un presupuesto neto anual incremental de 0,0007 USD PMPM, lo que representa una media del 5% del presupuesto total para el período de 5 años, mientras que una disminución del 20% en el precio del medicamento se traduciría en un ahorro presupuestario neto anual de -0,0001 USD PMPM, lo que representa un ahorro promedio del 0,75% del presupuesto anual. Se observaron variaciones menores para los demás parámetros. Para el sector privado, los resultados del análisis de sensibilidad determinístico fueron similares.

**Figura 2:** Análisis de sensibilidad determinista: impacto presupuestario promedio del PMPM desde la perspectiva de la seguridad social, USD.

## DISCUSIÓN

Con un costo estimado de 47 USD por unidad de 15 mg, la introducción del UPA + MTX implicaría un aumento mínimo de los costos para el sistema de salud en Argentina, lo que podría hacer que esta opción eficaz de tratamiento sea más accesible para los pacientes con artritis reumatoide. Por otro lado, los resultados del impacto presupuestario no superaron el umbral estimado para el alto impacto presupuestario en Argentina. Por lo tanto, nuestros hallazgos proporcionan evidencia sobre el impacto presupuestario a los pagadores que están considerando incorporar upadacitinib en su lista de medicamentos para tratar la AR, así como una herramienta para informar las negociaciones de precios basadas en el valor o los acuerdos de riesgo compartido. El costo de adquisición de los medicamentos para la artritis reumatoide representó el principal costo médico directo, lo que concuerda con otras investigaciones realizadas en el entorno local ^4^.

Calculamos el impacto presupuestario neto PMPM, que representa el costo o los ahorros adicionales para cada miembro cubierto por un plan de salud. Por ejemplo, si una institución con 2 millones de miembros formara parte del sector de seguridad social que reembolsara el tratamiento con upadacitinib, el aumento promedio total de los 2 millones de miembros sería de 0,0003 USD PMPM multiplicado por 2 millones de miembros, lo que equivale a 600 USD en aumento del presupuesto por mes. La introducción de esta nueva opción de tratamiento representa un aumento mínimo en los costos de un plan de salud grande, al tiempo que proporciona una terapia eficaz adicional para los pacientes con artritis reumatoide.

Según las directrices clínicas, los pacientes con RI-DMARc y factores de mal pronóstico pueden recibir varios productos biológicos y otras moléculas (anti-JAK) ^5^^-^^7^. Para los pacientes sin factores pronósticos desfavorables, pero con una respuesta inadecuada, las directrices recomiendan cambiar a o añadir otro DMARc (combinación de DMARc, recetar un DMARb o un anti-JAK) ^5^^-^^7^. No encontramos evaluaciones económicas de otras moléculas anti-JAK para el tratamiento de la AR en Argentina. Una evaluación de 2021 de los efectos económicos de upadacitinib en pacientes con artritis reumatoide realizada en los EE.UU. mostró que el tratamiento combinado con upadacitinib frente al tratamiento combinado con tofacitinib y la monoterapia con upadacitinib frente a la monoterapia con metotrexato se asociaron con costos médicos directos significativamente más bajos ^35^. Un análisis de minimización de los costos e impacto presupuestario de upadacitinib en el tratamiento de la AR en el Sistema Único de Salud (SUS) de Brasil durante un período de 5 años mostró que el upadacitinib tenía costos más bajos que el baricitinib y que tenía el costo más bajo entre los DMAR biológicos disponibles en el SUS; en comparación con nuestro análisis, de acuerdo a este estudio la introducción de upadacitinib supuso un ahorro neto ^36^.

Un estudio de costo-efectividad del tratamiento en pacientes con AR y RI-DMARc y en pacientes con una respuesta inadecuada a los DMARb anti-TNF demostró que el tofacitinib se utilizaba con frecuencia en pacientes con RI-DMARc y que se asociaba con un ahorro de costes (-337.489 €/año de vida ganado ajustado por calidad) ^37^. En los EE.UU., otro estudio mostró que una estrategia de tratamiento con tofacitinib reducía los costos y mejoraba la calidad de vida en comparación con otras estrategias de tratamiento ^38^. Una evaluación de los efectos económicos de tofacitinib, introducida tras una respuesta inadecuada al MTX o a uno o dos anti-TNF, mostró que el tofacitinib, como tratamiento de segunda o tercera línea, se asoció con costes más bajos que el tofacitinib en el contexto de una cuarta línea después del MTX y dos anti-TNF de forma secuencial. ^39^. En un análisis del impacto presupuestario y costo por respondedor para el tratamiento de la AR de moderada a severa, baricitinib fue una opción más costo-efectiva que otros DMAR con una eficacia comparable en pacientes con una respuesta inadecuada a los anti-TNF ^40^.

Nuestro estudio tiene varias limitaciones que deben reconocerse. Los resultados se basan en proyecciones de la cuota de mercado. Además, el uso de un modelo estático puede haber simplificado demasiado la historia natural de la enfermedad; sin embargo, nuestras evaluaciones se estructuraron en función de las recomendaciones de Mauskopf *et al*. ^21^. La falta de datos epidemiológicos locales fue otra limitación; por lo tanto, se pidió a los expertos locales que validaran los parámetros seleccionados. Además, es posible que el uso de precios de lista para fármacos biológicos y anti-JAK no refleje la situación real, ya que los pagadores pueden recibir descuentos y reembolsos bajo negociación de los fabricantes. Por último, teniendo en cuenta las condiciones macroeconómicas actuales en Argentina, se debe prestar especial atención a la evolución de los precios de los medicamentos. Este estudio tiene varias fortalezas. En primer lugar, creamos un modelo de impacto presupuestario que tiene en cuenta plenamente las características del sistema de salud y la práctica clínica en Argentina. En segundo lugar, la epidemiología, la utilización de los recursos, los datos de costos y las estimaciones se elaboraron en conjunto con expertos locales. Todos los costos de los eventos relacionados con la salud se calcularon mediante métodos de micro-costeo que incluyeron la identificación, la cuantificación, las tasas de utilización y los costos unitarios de los recursos de salud, lo que permitió realizar estimaciones sólidas de los parámetros de costos a nivel local. Finalmente, el modelo de impacto presupuestario incluyó un análisis de sensibilidad determinístico, que permitió dar cuenta de la incertidumbre de los resultados.

En conclusión, nuestros resultados podrían informar a los financiadores responsables de la asignación eficiente de los recursos presupuestarios y a otras partes interesadas e investigadores de la salud. Los estudios futuros que evalúen los beneficios de las tasas de remisión más altas de la AR en comparación con los costos derivados del tratamiento con nuevas moléculas contribuirían aún más a medir el impacto del uso de los recursos disponibles para la AR en Argentina. En el análisis de caso base y en la mayoría de los análisis de sensibilidad, upadacitinib supuso un incremento marginal de costes en el tratamiento de los pacientes con AR de moderada a severa y RI-DMARc.
